# Circulating immune cell profiling in advanced cervical and endometrial cancer patients treated with PD-1 blockade, radiotherapy, and immune modulation in the PRIMMO trial

**DOI:** 10.3389/fimmu.2026.1794131

**Published:** 2026-05-21

**Authors:** Regina Esi Mensimah Baiden-Amissah, Sandra Tuyaerts, Daniela Annibali, Alejandro Herreros-Pomares, Wout De Wispelaere, Rossana Maria Benedetto, Emiel A. De Jaeghere, An M. T. Van Nuffel, Peter Vuylsteke, Stéphanie Henry, Xuan Bich Trinh, Peter A. Van Dam, Sandrine Aspeslagh, Alex De Caluwé, Eline Naert, Katrien Vandecasteele, Hannelore G. Denys, Frédéric Amant

**Affiliations:** 1 Gynecologic Oncology, Department of Oncology, Katholieke Universiteit Leuven (KU Leuven), Leuven, Belgium; 2 Leuven Cancer Institute, Leuven, Belgium; 3 Department of Medical Oncology, University Hospital Brussels, Brussels, Belgium; 4 Translational Oncology Research Center (TORC), Vrije Universiteit Brussel, Brussels, Belgium; 5 Gynecological Oncology, The Netherlands Cancer Institute, Amsterdam, Netherlands; 6 Department of Medical Oncology, Ghent University Hospital, Ghent, Belgium; 7 Cancer Research Institute Ghent (CRIG), Ghent, Belgium; 8 Anticancer Fund, Strombeek-Bever, Belgium; 9 Department of Hemato-Oncology, Centre Hospitalier Universitaire, Université Catholique de Louvain Namur (Sainte-Elisabeth), Namur, Belgium; 10 Department of Gynecologic Oncology and Senology, University Hospital Antwerp, Edegem, Belgium; 11 Multidisciplinary Oncologic Centre Antwerp (MOCA), University Hospital Antwerp, Edegem, Belgium; 12 Center for Oncological Research (CORE), Integrated Personalized and Precision Oncology Network (IPPON), Edegem, Belgium; 13 Université Libre de Bruxelles (ULB), Hôpitaux Universitaires de Bruxelles (H.U.B), Institut Jules Bordet, Brussels, Belgium; 14 Department of Radiation Oncology, General Hospital Sint-Maarten, Mechelen, Belgium; 15 Department of Radiation Oncology, Ghent University Hospital, Ghent, Belgium; 16 Center for Gynecologic Oncology Amsterdam (CGOA), Netherlands Cancer Institute and Amsterdam Medical Center, Amsterdam, Netherlands; 17 Department of Gynecology and Obstetrics, University Hospitals Leuven, Leuven, Belgium

**Keywords:** anti-PD-1 therapy, blood-based biomarkers, combination therapy, cyclophosphamide, drug repurposing, gynecological cancers, immune checkpoint blockade, radiotherapy

## Abstract

**Background:**

Immune checkpoint blockade (ICB) has revolutionized cancer treatment, including gynecological cancers, yet durable responses are observed only in subsets of patients. To improve efficacy, ICB is increasingly combined with complementary approaches. Although tissue-based biomarkers provide valuable information, their invasiveness and inability to capture systemic immune changes limit their utility. Peripheral blood offers a noninvasive alternative, but immune cell profiling in advanced cervical cancer (CC) and endometrial (EC) patients receiving ICB-based therapy remain underexplored.

**Methods:**

This exploratory translational study of the PRIMMO clinical trial (https://clinicaltrials.gov/search?id=%22NCT03192059%22) analyzed blood-based immune profiles from advanced CC (n=19) and EC (n=24) patients treated with an ICB-based regimen. Peripheral blood was collected at inclusion (baseline), on-treatment (week 7), and post-treatment (week 26 or earlier (< week 26) in case of earlier disease progression). Immune cell subsets and selected systemic immune mediators were assessed by multicolor flow cytometry and ELISA.

**Results:**

At baseline, CTLA-4^+^PD-1^+^CD4^+^ T cells and CD161^+^CD56^+^CD16^+^ NK cells were associated with survival outcomes only, while the combined immune score was associated with both treatment response and survival outcomes. During treatment, responders exhibited relatively stable immune profiles at both early and late treatment time points. In contrast, non-responders showed sustained decreases in pDCs and increases in activation (CD69, CD137, HLA-DR) and co-stimulatory/inhibitory markers (CTLA-4, ICOS, Tim-3) across T cell subsets, including co-expression at both early and late phases of treatment. NK cell subsets also displayed increased activation features (CD69, CD161, HLA-DR). These changes were accompanied by expansion of immunoregulatory populations (MDSCs and Tregs), quantified based on phenotypic markers only, along with an increased kynurenine/tryptophan ratio, and elevated sPD-1 levels. Tumor-type-specific analyses suggested relatively more NK-related changes in CC and T cell-related changes in EC.

**Conclusion:**

Longitudinal immune changes, rather than baseline differences alone, distinguished responders from non-responders. Non-response to the combination treatment is not characterized by immune inactivity but rather associated with sustained and coordinated increases in both activation and regulatory immune features, suggesting a shift toward systemic immune imbalance following treatment. These findings support the need for longitudinal, integrative immune monitoring strategies in future clinical trials.

## Graphical Abstract

**Figure:**
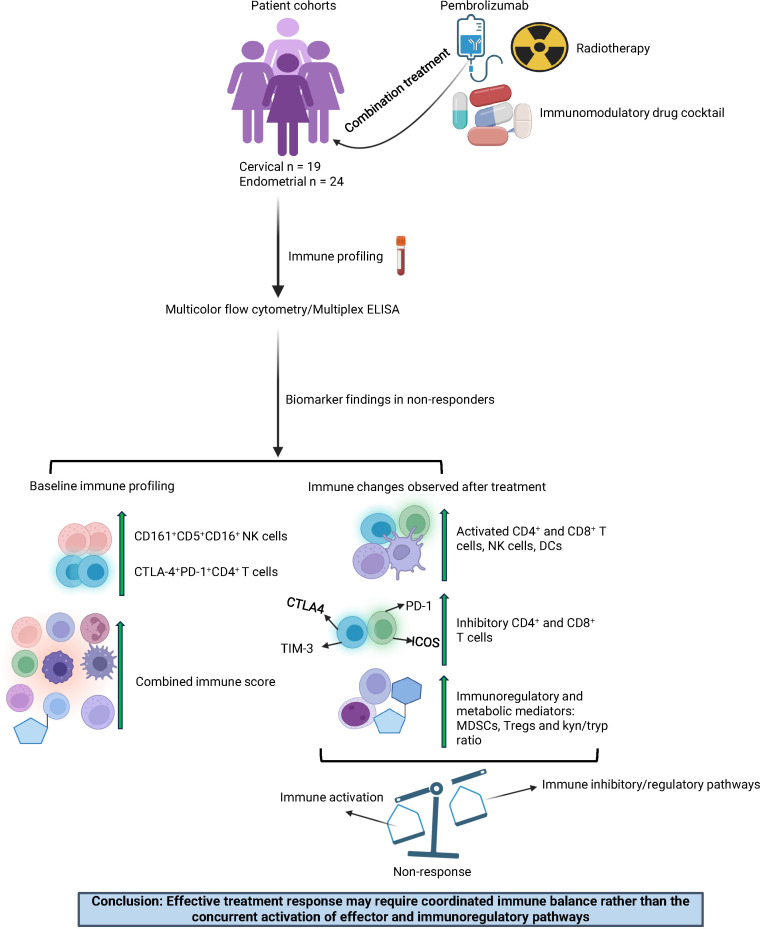

## Introduction

Cervical cancer (CC) and endometrial cancer (EC) represent two of the most prevalent gynecologic malignancies worldwide. In 2022, CC accounted for an estimated 660,000 new cases and about 350,000 deaths, ranking fourth among female cancers, whereas EC represented the sixth most common malignancy, with roughly 420,000 new diagnoses and 98,000 deaths globally (1).

The introduction of immune checkpoint blockade (ICB), especially antibodies targeting the programmed cell death protein (PD) and its ligand PD-L1, has transformed cancer immunotherapy. However, anti-PD-(L)1 monotherapy has produced modest responses in advanced CC and EC compared to more immunogenic tumors such as melanoma or non-small-cell lung cancer. Reported objective response rates (ORRs) to single-agent anti-PD-(L)1 are approximately 26% in EC, with higher efficacy in mismatch repair-deficient/microsatellite instability-high (dMMR/MSI-H) cases (44%) (2). In CC, ORRs to single-agent anti-PD-1 range between 16-29% in PD-L1-positive recurrent or metastatic cases (3, 4). These limited responses emphasize the crucial role of the tumor microenvironment (TME) in regulating immune resistance and disease progression (5). Developing a combinatorial approach that positively modulates the TME has the potential to significantly improve immunotherapy efficacy (6).

Recent clinical advances have positioned ICB-based combinations as an integral component of therapy for both CC and EC approved by the U.S. Food and Drug Administration (FDA) and/or the European Medicines Agency (EMA). In CC, ICBs are integrated into first-line treatment strategies, often in combination with chemotherapy, with or without anti-angiogenic agents, or concurrently with chemoradiation in selected locally advanced cases. In later settings, ICB monotherapy is used for recurrent or metastatic disease, with treatment decisions in some cases guided by tumor immune biomarker expression and in others administered irrespective of biomarker status (7, 8). In EC, first-line approaches frequently combine ICBs with chemotherapy, often followed by maintenance ICB, providing a frontline systemic approach for advanced or recurrent diseases. In later settings, ICBs are used either as monotherapy for tumors with MMR/MSI status, or in combination with targeted agents for tumors lacking these molecular features (9, 10).

Despite the clinical success of ICBs, predictive biomarkers that can reliably identify patients most likely to benefit remain limited. Currently, PD-L1 expression, dMMR/MSI status, and tumor mutational burden (TMB) are the only FDA- and/or EMA-approved biomarkers for ICB selection. While valuable, their predictive power remains incomplete, even for robust markers such as dMMR/MSI (11–14). Furthermore, tissue-based assays are invasive and difficult to perform longitudinally due to practical and ethical limitations. Even with serial biopsies, sampling bias from spatial heterogeneity and missed temporal changes in the tumor microenvironment (TME) may limit their ability to fully capture tumor and immune dynamics over time. These limitations have spurred growing interest in developing non-invasive, blood-derived biomarkers, including circulating immune cell populations, which may reflect systemic tumor-immune interactions and enable real-time monitoring of therapeutic efficacy. A growing body of evidence across multiple solid tumors, including lung cancer, melanoma and gastric cancer, highlights the importance of immune dynamics during treatment and supports the predictive and prognostic relevance of distinct peripheral immune subsets in patients receiving ICB (15–19). However, until now the investigation of circulating immune cells and their longitudinal evolution under treatment remain insufficiently characterized in advanced CC and EC is limited.

To improve the efficacy of pembrolizumab through modulation of the TME, the PRIMMO trial evaluated a combination of pembrolizumab, radiotherapy, and an immunomodulatory drug cocktail (IDC) consisting of low-dose cyclophosphamide, and repurposed drugs (aspirin, lansoprazole, vitamin D, and curcumin) in patients with advanced CC (n=19) or EC (n=24) (20). Radiotherapy can promote anti-tumor immunity through immunogenic cell death (21, 22). However, radiotherapy can exert suppressive effects, such as attracting Tregs and MDSCs to the TME (23) Each IDC component was selected for its potential to counter these effects: cyclophosphamide possesses both anti-angiogenic and anti-proliferative properties and can reduce Treg quantity and quality, thereby enhancing anti-tumor immunity (24, 25); vitamin D has been shown to potentiate NK cell function, reduce MDSC levels and increase tumor immune cell infiltration (26); aspirin inhibits cyclooxygenase (COX) enzymes activity, normalizes tumor vasculature and may reprogram tumor inflammatory milieu to support immune-mediated tumor control (27, 28); lansoprazole, may reduce acidity in the TME and was hypothesized to improve intratumoral immune cell function (29, 30); and curcumin has been shown to sensitize tumors to radiation and enhance the immunomodulatory effects of radiation (20, 31). Repurposed drugs such as vitamin D, aspirin and curcumin may synergize with anti-PD-(L)1 (32–34), although potential adverse effects, including microbiome disruption and increased immune-related toxicity, warrant further evaluation (35–37).

Although the trial did not meet its primary endpoint, early and durable clinical responses were documented in a subset of heavily pretreated patients with limited treatment options, with best overall response rate (BORR) of 22.2% in CC and 12.0% in EC (38). Importantly, the PRIMMO trial was accompanied by translational research aimed at characterizing immune-related biomarkers and the gut microbiome. Recently published exploratory analyses of the gut microbiome revealed associations between microbiome composition and clinical outcomes in CC and EC patients treated with the combination regimen. These findings provide novel mechanistic insights into host-immune interactions and suggest that microbiome-related factors may modulate responsiveness to ICB-based therapies in these malignancies (39). Systematic and longitudinal assessment of immune parameters associated with clinical benefit may further help improve the understanding of the limited responses observed in many patients in our study.

Here, we present exploratory translational immune analyses from the PRIMMO trial. The primary aim was to characterize baseline immune profiles in patients with CC and EC, and to investigate treatment-associated immune dynamics in relation to response. To this end, immune cell subset phenotyping and systemic immune mediator quantification were performed in peripheral blood at baseline, week 7, and end of treatment (EOT), and correlated with clinical outcome.

## Patients, materials and methods

### Study design and ethical approval

**Figure 1:**
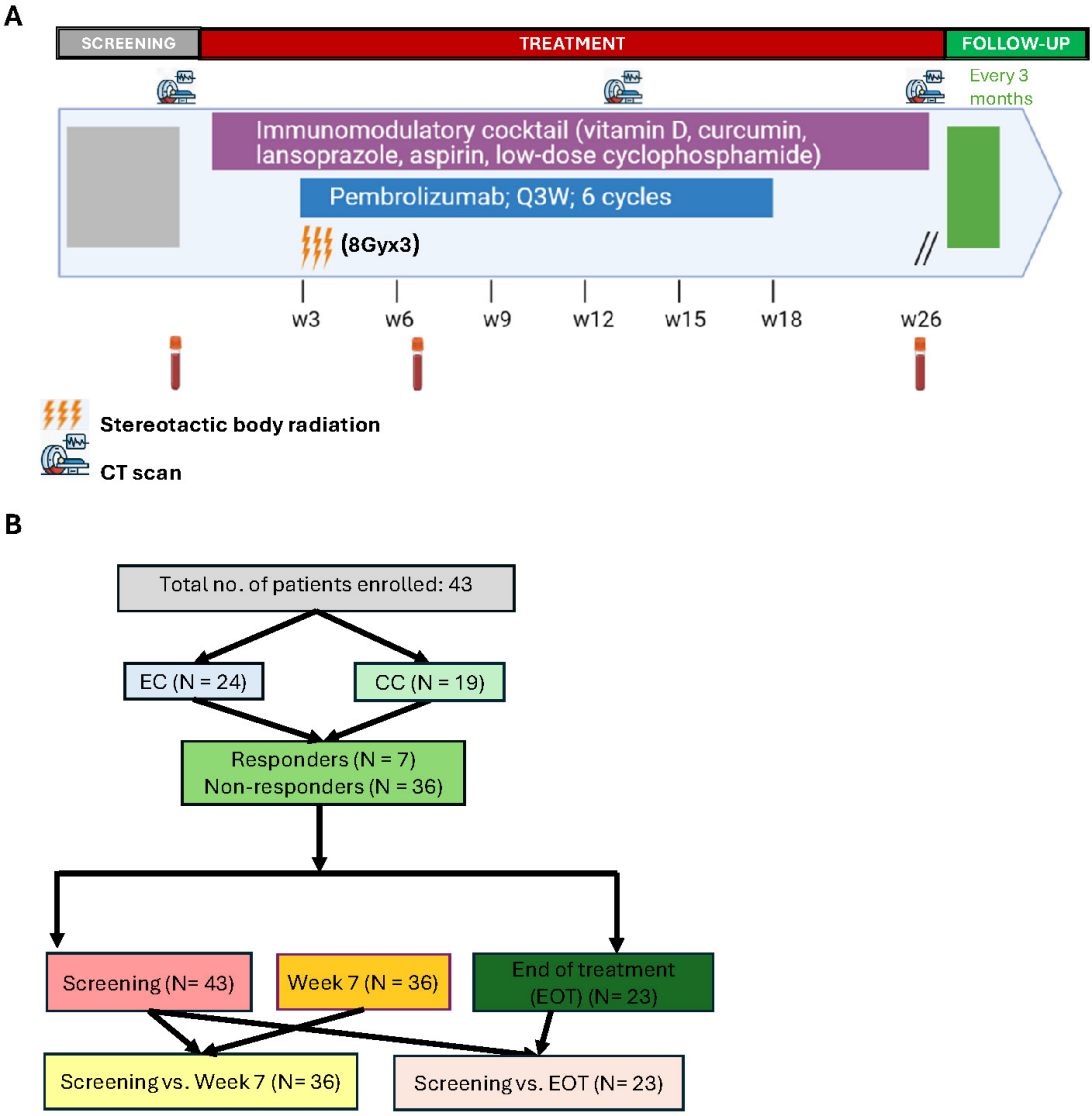
PRIMMO trial study design and sampling flowchart: **(A)** Cervical and Endometrial cancer patients were treated as indicated. Blood samples were taken at screening, week 7 and week 26/EOT (week 26 for responders and<week 26 for early progressors). **(B)** Patient enrollment per cohort and sample counts at screening, week 7, EOT and for paired comparisons (screening vs. week 7; baseline vs. EOT). CC, Cervical cancer; EC, Endometrial cancer; CT, computed tomography.

The PRIMMO clinical trial was a non-randomized, open-label, multicohort, non-comparative, multisite, phase II study with a safety run-in that was designed to assess the efficacy and toxicity of pembrolizumab, stereotactic body radiotherapy (SBRT) (8Gyx3), and an IDC in unselected patients with persistent/recurrent/metastatic CC or EC. The trial adhered to the Declaration of Helsinki and the International Council for Harmonization Guidelines for Good Clinical Practice and obtained approval from the independent ethics committee at each participating institution (central committee: Ghent University Hospital Ethics Committee, identifier EC/2017/0304). Prior to treatment initiation, all participants provided written informed consent for immune biomarker analysis. Details of the study protocol, including patient population and eligibility criteria, study regimen and clinical assessments, has previously been published (20). For this translational study, samples were obtained from 19 CC and 24 EC patients. The sample size was predefined according to the clinical trial design and was not specifically powered for the translational analyses. Accordingly, translational analyses are exploratory, and no formal power calculations were performed for the translational endpoints. For immune monitoring, blood was drawn at baseline (screening), on-treatment (week 7), and post-treatment (week 26 or earlier (< week 26) in case of earlier disease progression). The treatment schedule and sampling time points are indicated in **Figure 1A**.

### Blood sampling and processing

For serum preparation, blood was collected at different time points in silica-treated tubes (serum separating tubes) and centrifuged for 10 minutes at 1300g at room temperature (RT). The resulting supernatant (serum) was immediately collected, divided into 0.2- and 0.5-mL aliquots and stored at -80 C until analysis.

For plasma, blood was collected at different time points in EDTA/heparin tubes and centrifuged for 10 minutes at 1800 rpm at RT. The plasma was then collected and transferred into a new tube and centrifuged again for 10min at 800g at RT. The resulting supernatant (plasma) was immediately divided into 0.2- and 0.5-mL aliquots and stored at -80 C until analysis.

PBMCs were isolated from heparin tubes, cryopreserved, and thawed as previously described (38).

### Flow cytometry

Briefly, thawed PBMCs were quickly washed and counted manually with trypan blue (Merck, UK). Cell number was adjusted to a minimum concentration of 5.0x10^5^ cells/ml in ice cold FACS buffer (PBS, 0.5% BSA). Cells were stained with a Fixable Viability Dye eFluor™ 506 (Thermo Fisher Scientific, USA) for 30min at 4°C, followed by Fc receptor blocking with 10% goat serum (Merck, UK). Next, cells were stained with different panels of fluorescently conjugated anti-human monoclonal antibodies and incubated for 30min at 4°C (**Supplementary Table 1**). For intracellular staining, cells were fixed and permeabilized using the Fixation/Permeabilization Kit (Thermo Fisher Scientific, USA). FoxP3 was stained for the Treg panel, and arginase-1 was stained for the MDSC panel. MDSC subsets were analyzed directly in whole blood samples, as polymorphonuclear MDSC (PMN-MDSC) cells were removed following Ficoll density gradient separation. Appropriate isotype control antibodies and fluorescence-minus-one control stains were used to perform gating. Samples were acquired on BD FACS Canto II cytometer (BD Biosciences, USA) and analyzed with FlowJo software, version 10.6.2 (BD Biosciences, USA). Subgroups of cells were quantified as a percentage of live cells of the respective cell type (e.g., T cell, NK cell, MDSC, dendritic cell (DC)). Activation and inhibitory marker expression was calculated as the percentage of cells expressing a specific marker within a specific cell subgroup. The gating strategies for the different immune cell types are described in the supplementary data (**Supplementary Figures 1**-**4**).

### Analysis of plasma and serum

Arginase-1 activity in plasma was determined by measuring the conversion of ʟ-arginine to ʟ-ornithine and urea using the QuantiChrom Arginase assay kit according to manufacturer’s instructions (Merck, USA). Optical density (OD) was measured at 450 nm with a Multiskan FC reader (Thermo Fisher Scientific, USA). Arginase-1 activity was calculated as indicated in the manufacturer’s instructions.

Kynurenine (Kyn) and tryptophan (Tryp) levels in serum were detected using Kynurenine and Tryptophan enzyme-linked immunosorbent assay (ELISA) kits respectively according to manufacturer’s instruction (LDN, Germany). OD at 450 nm was measured and concentrations were calculated from a standard curve.

A custom LEGENDplex assay was used to measure the levels of sPD-1, sPD-L1 and sPD-L2 in patients’ serum according to manufacturer’s instructions (Biolegend, USA). Samples were acquired on a BD FACS Canto II cytometer (BD Biosciences, USA). Forward scatter (FSC) and side scatter (SSC) were used to identify beads A and B, fluorescence in the APC channel further sub-classified the beads for each analyte and fluorescence intensity in the PE channel is a measure for the analyte concentration, which is calculated from a standard curve. LEGENDplex QOGNIT software (Biolegend) was used for data analysis.

### Data analysis of immune parameters in patients’ peripheral blood

To assess the immune profiles or changes in patients over the course of treatment, we employed multicolor flow cytometry and ELISA, analyzing circulating immune cell subsets and systemic immune mediators, respectively. Immune cell subsets were initially classified based on lineage-specific markers (CD8, CD4, NK and DC). Their phenotypic profile was subsequently assessed by evaluating the expression of activation and inhibitory markers. Systemic immune mediators and distinct immune cell subsets (including expression of activation and inhibitory markers) are collectively referred to as immune cell markers (ICMs) in this study. Analyses were conducted at three key timepoints: screening (n=43), week 7 (n=36), and end of treatment (EOT, n=23). Of the EOT samples, 10 were obtained from patients who completed the full treatment course at week 26, while the remaining 13 were from patients who experienced earlier disease progression and exited the study prior to week 26 (<Week 26). A comprehensive list of the phenotypic markers used for immune subset identification is provided in **Supplementary Table 2**. ICMs were examined at each timepoint and correlated with the clinical outcome of the patients. Furthermore, temporal changes in these ICMs were assessed by comparing longitudinal samples from screening to week 7 (n=36; 7 responders and 29 non-responders), and from screening to EOT (n=23; 7 responders and 16 non-responders). Patient enrollment and detailed sample collection workflow are summarized in **Figure 1B**, **Supplementary Table 3**.

### Statistical analysis

Differences between independent groups (responders vs. non-responders, cervical vs. endometrial tumors and early progressors vs. responders) were assessed using the Mann-Whitney U test. Paired comparisons (screening vs. week 7 and screening vs. end of treatment [EOT]) and (screening vs. week 7 vs. EOT) were performed using the Wilcoxon matched-pairs signed-rank test and Friedman Test respectively. To control for multiple testing, p-values were adjusted using the Benjamini-Hochberg false discovery rate (FDR) method. Unsupervised hierarchical clustering was performed in R (v4.0.3) using the pheatmap (v1.0.12), gplots (v3.1.3.1), and RColorBrewer (v1.1-3) packages (Bioconductor v3.18). Correlations between continuous variables were evaluated using Spearman’s rank correlation coefficient.

Survival analyses were conducted using Cox proportional hazards regression models. Univariate models were used to assess associations between individual ICMs, clinical variables, and survival outcomes, with hazard ratios (HRs) reported with 95% confidence intervals (CIs) and corresponding p-values. Variables with p< 0.05 in univariate analyses were included in multivariate models for overall survival (OS) and progression-free survival (PFS). Clinically relevant covariates (tumor type and tumor grade) were included irrespective of statistical significance.

A combined immune response score was developed based on the discriminative ability of individual ICMs. Comparisons between receiver operating characteristic (ROC) curves were performed using the method of DeLong et al. (40), and 95% confidence intervals for the area under the curve (AUC) were calculated according to Hanley and McNeil (41). To reduce overfitting, only markers with consistent directionality and AUC ≥ 0.6 or ≤ 0.4 were included. For each of the 13 selected ICMs, an optimal cutoff value was determined using Youden’s index derived from ROC curves based on baseline (screening) measurements. Markers with AUC ≥ 0.6 were dichotomized per patient as above (1) or below (0) the cutoff. Markers with AUC ≤ 0.4 were inversely coded to ensure that both direct and inverse associations with response contributed to the final score. Markers with stronger discriminative ability (AUC ≥ 0.7 or ≤ 0.3) were assigned double weight, as previously described (42, 43). The final immune response score for each patient was calculated as.


$$Immune\;response\;scor\mathrm{e =}\;\frac{\sum \left(all\;13\;immune\;cell\;markers\times weight\right)}{maximal\;weight}\times 100$$

Given the exploratory nature of the model, the weighting of individual markers was predefined and should be interpreted as hypothesis-generating. The baseline combined immune score was derived *post hoc* and was neither trained on nor validated in independent cohorts. Model calibration was assessed by plotting predicted versus observed probabilities using 500 bootstrap resamples. Although bootstrap resampling does not replace external validation, it provides a robust internal assessment when independent datasets are unavailable. Discriminative performance was evaluated by estimating an optimism-corrected AUC using 1,000 bootstrap replicates.

All statistical analyses were performed using GraphPad Prism (v8.4.3) and R (v4.0.3). All tests were two-sided, and p-values ≤ 0.05 were considered statistically significant.

## Results

### The combined treatment in the PRIMMO study demonstrated a modest but durable anti-tumor effect in cervical and endometrial cancer patients

The PRIMMO study enrolled 19 CC and 24 EC patients. All patients had recurrent and/or refractory disease, and 50% had received one prior systemic therapy for advanced disease, while the others had received two or more. The best overall response rate (BORR) by RECIST was 22.2% (90% CI 8.0-43.9) in the CC cohort and 12.0% (90% CI 3.4-28.2) in the EC cohort. In total, 7 patients responded, (CC, n = 4 and EC, n = 3) classified as either partial response (PR) or complete response (CR), while 36 patients (CC, n = 14 and EC, n = 22) were non-responders, exhibiting either stable disease (SD) or progressive disease (PD). Median irPFS was 3.6 weeks (95% CI, 3.6-15.4) for EC and 4.1 weeks (95% CI, 4.1-25.7) for CC. Median OS was 37.4 weeks (95% CI, 19.0-50.3) and 39.6 weeks (95% CI, 15.0-67.0) in EC and CC, respectively (38). **Supplementary Table 4** depicts detailed patient characteristics.

### Baseline immune landscape and association with clinical outcomes

**Figure 2:**
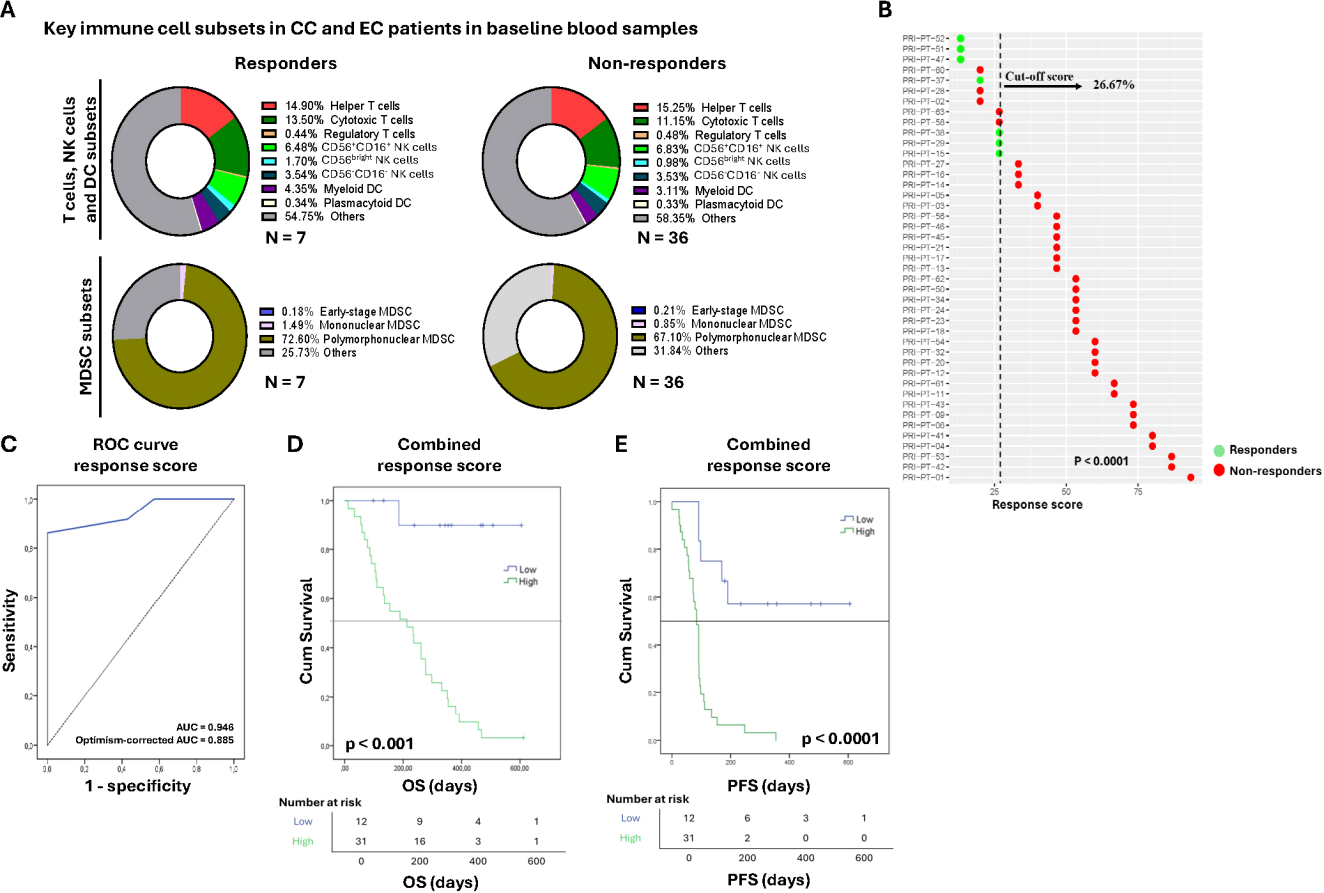
Baseline immune profiling and association with clinical outcomes in cervical and endometrial cancer patients: **(A)** Pie charts showing percentage of T cell, NK cell and DC subsets in PBMCs and MDSC subsets in whole blood in responders and non-responders at screening, percentages represent median values of each subpopulation; Best overall response rate (BORR) according to imaging at week 26 was used to differentiate responders (n = 7) from non-responders (n= 36). **(B)** Combined immune mediators score for discriminating treatment response in cervical and endometrial cancer patients: responders (green), non-responders (red). The optimal threshold to distinguish responders from non-responders (26.67%) was selected based on the value maximizing the ROC AUC of the response score, providing the best separation between groups; **(C)** ROC curve for prediction of combined response score; Significance difference was tested by Chi-square test **(D, E)** Kaplan-Meier analysis of **(D)** OS and **(E)** PFS outcome in patients with above versus below the cut-off value for combined immune score; Significance difference was tested by log-rank test. Significance difference was accepted at p ≤ 0.05.

To evaluate the immune status of patients prior to treatment, we analyzed key immune cell subsets in baseline peripheral blood samples using a panel of antibodies summarized in **Supplementary Table 1**. Notably, although the distribution of most immune subsets fell within established reference ranges, the frequency of total T cells was ~30%, substantially lower than the typical reference range of 45-70% (44) (**Figure 2A**). We did not observe differences between these key immune cell subsets between responders and non-responders. We then analyzed all ICMs (**Supplementary Table 2**) in baseline blood samples to explore their association with tumor type and treatment response. To obtain an overview of global patterns, we first performed an unsupervised clustering analysis, which revealed substantial inter-patient variability in cell type abundance and marker expression, without clear separation by tumor type or treatment response (**Supplementary Figure 5**). Building on these observations, we further explored potential subgroup differences in ICM profiles. We initially compared patients with CC and EC. No statistically significant differences were observed, suggesting similar ICM distributions across both tumor types (**Supplementary Table 5**). We then extended this analysis to assess the discriminatory capacity of individual ICMs in the overall cohort. To do this, we calculated the area under the receiver operating characteristic curve (AUC-ROC) using the original values of each ICM at screening and selected those with AUC values ≤ 0.4 (associated with favorable prognosis) or ≥ 0.6 (associated with poor prognosis). This approach identified 13-ICMs of interest (**Supplementary Table 6**).

We next investigated whether any of the 13-ICMs could independently distinguish responders from non-responders when evaluated as single biomarkers. No significant differences were observed between responders and non-responders for any individual ICM, suggesting that single ICM were not discriminatory for treatment response in this cohort. However, when evaluating survival outcomes, univariate and multivariate regression identified two ICMs with prognostic value. Univariate Cox proportional hazards models were used to assess associations between ICMs and clinical variables with OS and PFS (**Supplementary Tables 7**, **8**). Eight ICMs were significantly associated with survival outcomes in univariate analyses and were subsequently included in multivariate models together with clinically relevant covariates (tumor type and tumor grade). In multivariate Cox regression analyses, only CTLA-4^+^PD-1^+^ expression in CD4^+^ T cells remained significantly associated with OS, while CD161 expression in CD56^+^CD16^+^ NK cells remained significantly associated with PFS, indicating that higher expression levels of these markers were independently associated with worse prognosis (**Supplementary Table 9**).

To further explore their joint behavior, we constructed a combined immune score reflecting the overall discriminative capacity of these 13-ICMs between response groups. Briefly, ICMs were dichotomized according to ROC curves Youden’s index. ICMs with AUC ≥ 0.7 or AUC ≤ 0.3 were weighted double. To each patient, the combined response score was calculated as follows:


$$Immune\;response\;score=\;\frac{\begin{array}{c} \sum (eMDSC\times 0.1+sPDL1\times 0.2+CD161^{+}CD56^{+}CD16^{+}\times 0.1+HLADR^{+}CD56^{-}CD61^{+}\times 0.1+CD69^{+}CD56^{bright}\times 0.2+CD69^{+}CD137^{+}CD4^{+}\times 0.1+PD1^{+}CD4^{+}\times 0.1\\ +PD1^{+}CTLA4^{+}CD4^{+}\times 0.1+CD69^{+}HLADR^{+}CD8^{+}\times 0.1+CD11c^{+}CD123^{-}\times 0.1+CD56^{+}CD16^{+}\times 0.1+CD56^{bright}\times 0.1+CTLA4^{+}ICOS^{+}CD8^{+}\times 0.1) \end{array}}{1.5}\times 100$$

A cut-off of 26.67% was obtained, identifying patients with a value ≤ 26.67% as potential responders and those with a value > 26.67% as potential non-responders (**Figure 2B**). A Mann-Whitney U test further showed that the combined score significantly separated responders from non-responders (p< 0.0001). Its discriminative performance was further supported by an AUC of 0.946 (p< 0.0001, Chi-square test). Internal validation using bootstrap resampling showed a mean optimism of 0.061 (**Supplementary Figure 6**), resulting in an optimism-corrected AUC of 0.885 (**Figure 2C**). Additionally, Kaplan-Meier analyses showed that patients with a score ≤ 26.67% experienced improved OS and PFS, compared to those with a score > 26.67% (**Figures 2D, E**). To evaluate whether these associations were independent of clinical covariates (tumor grade and tumor type), multivariate analyses were performed and HRs, 95% CIs, and p-values were calculated. The combined score remained an independent prognostic factor for both OS (HR 19.040, 95% CI: 2.572-140.957, p = 0.004) and PFS (HR 7.079, 95% CI: 2.613-19.176, p< 0.001) (**Supplementary Table 10**).

### Longitudinal changes in immune cells and soluble immune mediators during treatment are associated with clinical outcomes and tumor type

Next, we examined changes in ICMs over time and their association with clinical outcomes by analyzing blood samples collected at screening, week 7, and EOT. ICMs were compared between responders and non-responders at week 7 and EOT, and longitudinal analyses were performed to assess changes during early (screening vs. week 7) and late (screening vs. EOT) treatment phases. Analysis of key immune cell subsets at week 7 and EOT revealed no statistically significant changes between responders and non-responders. (**Supplementary Figures 7A, B**). Consistent with observations at screening, no significant differences were observed between responders and non-responders across all assessed ICMs, including key immune cell populations at either week 7 or EOT timepoints (data not shown).

**Figure 3:**
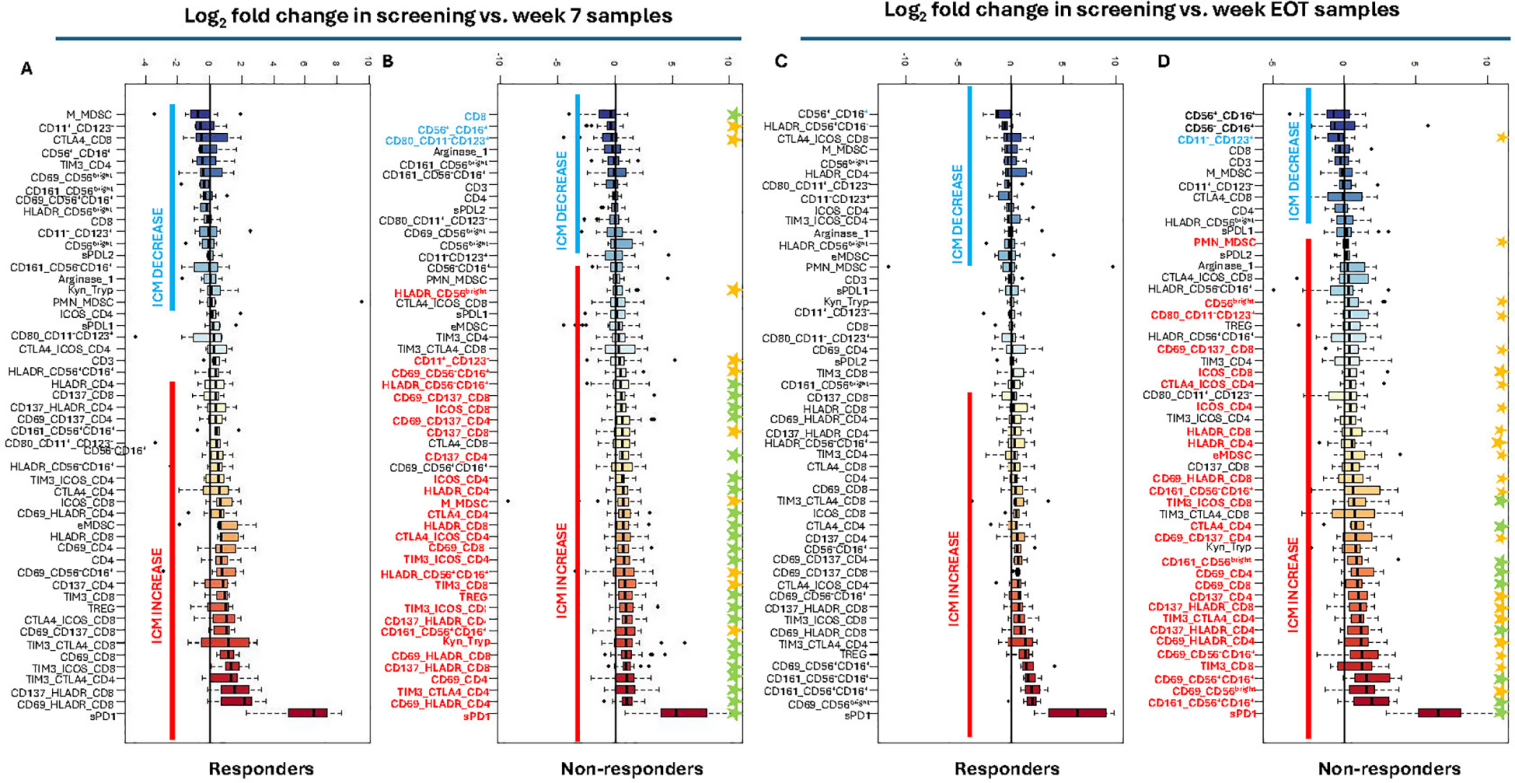
Alterations of immune cells and systemic immune mediators during treatment were associated with clinical benefits in cervical and endometrial cancer patients: **(A, B)** Fold change of soluble mediators and cells expressing activation and inhibition markers in **(A)** responders and **(B)** non-responders at screening versus week 7. **(C, D)** Fold change of soluble mediators and cells expressing activation and inhibition markers in **(C)** responders and **(D)** non-responders at screening versus EOT (week 26 for responders and<week 26 for early progressors). The results shown are the log 2 of the ratio between the percentage of cells expressing makers at screening vs. week 7 (N=36) and screening vs. EOT (N=23). Asterisks indicate p< 0.05 (yellow) and p< 0.01 (green). Significance difference was tested by Wilcoxon signed-rank test, with multiple comparison using the Benjamini–Hochberg false discovery rate (FDR) procedure. Best overall response rate (BORR) according to imaging at week 26 was used to differentiate responders (n = 7) from non-responders (n= 36). Significance difference was accepted at p ≤ 0.05. Blue and red lines represent general decreases and increases in ICMs during treatment, respectively, regardless of whether these changes were statistically significant. ICMs in the upper panels (blue lines) reflect decreases from baseline, while those in the lower panels (red lines) reflect increases.

Because screening, week 7, and EOT samples were not consistently available from the same patients, in the non-responding group, longitudinal analyses were limited to comparisons between screening and week 7 and between screening and EOT. Although incomplete data from non-responders limited the ability to fully characterize their longitudinal immune profiles, their exclusion does not affect the interpretation of trends observed among responders. Longitudinal analyses identified significant changes in ICMs between screening vs. week 7 and screening vs. EOT, and their association with clinical response was subsequently evaluated (**Figures 3A–D**). ICMs containing PD-1 were excluded from the analysis because pembrolizumab binds directly to PD-1 to achieve its therapeutic effect, thereby leading to decreased PD-1 detection upon treatment and rendering evaluation of PD-1 changes irrelevant.

Comparison of immune cell profiles between screening and week 7 revealed relatively stable immune profiles among responders (n=7) (**Figure 3A**). In contrast, non-responders (n=29) exhibited more pronounced alterations in multiple ICMs during early phase of treatment. (**Figure 3B**, **Supplementary Table 11**). Within the T cell compartment, CD4^+^ and CD8^+^ T cells in non-responders showed elevated expression of activation markers CD69, CD137 and HLA-DR, both individually and in co-expression patterns. Non-responders also displayed elevated levels of co-stimulatory marker ICOS, as well as inhibitory markers Tim-3 and CTLA-4 on CD4^+^ and CD8^+^ T cells, with notable co-expression. Additionally, they showed reduced frequencies of CD8^+^ T cells and increased frequencies of Tregs. In the NK cell compartment, non-responders exhibited decreased frequencies of CD56^+^CD16^+^ NK cells, accompanied by increased expression of CD161 and HLA-DR on these cells. CD56^-^CD16^+^ NK cells were also elevated, displaying expressions of CD69 and HLA-DR, as well as CD56^bright^ NK cells marked by HLA-DR expression. Beyond lymphocytes, non-responders exhibited significant immunological alterations in systemic immune mediators and myeloid populations. Levels of kyn/tryp ratio, sPD-1, M-MDSCs and mDC were all elevated, whereas CD80 expression on pDC was reduced (**Figure 3B**, **Supplementary Table 11**).

Longitudinal analysis between screening and EOT samples mirrored some of the trends observed at screening vs. week 7. Similar to the early phase, immune profiles in responders remained stable (**Figure 3C**)). Conversely, non-responders demonstrated increased levels of CD56^bright^ and CD56^+^CD16^+^ NK cells expressing activation markers CD69 and CD161, as well as elevated levels of sPD-1, e-MDSCs, accompanied by a reduction pDC. Moreover, non-responders showed increased frequencies of both CD4^+^ and CD8^+^ T cells expressing the following markers: CD69, CD137, HLA-DR, CTLA4, ICOS, TIM3, including co-expression profiles (**Figure 3D**, **Supplementary Table 12**). A detailed summary of immune cell population changes, in non-responders, is provided in **Supplementary Tables 11**, **12**.

In addition to treatment response in the overall cohort, we also assessed whether immune changes differed according to tumor type. During treatment, significant changes in distinct immune cell subtypes between tumor types were observed. In the early phase, patients with CC showed predominantly increased CD56^+^CD16^+^ and CD56^-^CD16^+^ NK cells expressing activation markers (CD69, CD161, and/or HLA-DR), whereas patients with EC exhibited increases in CD4^+^ and CD8^+^ T cells expressing CD69, CD137, and/or HLA-DR (**Supplementary Table 13**). In the late treatment phase, CC was characterized by increased CD56^+^CD16^+^ and CD56^-^CD16^+^ NK cells expressing activation markers (CD69, CD161, and/or HLA-DR), as well as CD4^+^ T cells expressing CD69, CD137, HLA-DR, CTLA-4, and ICOS, accompanied by an elevated kyn/tryp ratio and increased sPD-L2 levels. In contrast, late treatment in EC was marked by increased CD8^+^ T cells expressing CD69, CD137, and/or HLA-DR, as well as Tim-3 and ICOS (**Supplementary Table 14**).

### Longitudinal immune changes during treatment in early progressors and responders

As per protocol, peripheral blood samples were scheduled for collection at screening, week 7 and week 26. However, a subset of patients (n=13) experienced rapid disease progression and were unable to continue treatment till week 26. For these patients, EOT (< week 26) samples were collected at the time of progression (range: 6.2-19.6 weeks; median 13.65 weeks). To explore potential immune correlates of early progression to the combination therapy, we assessed ICMs in EOT samples (< week 26) from these early progressors (ePR) and compared them with those from patients who completed treatment and exhibited a clinical response at Week 26 (R, n=7).

**Figure 4:**
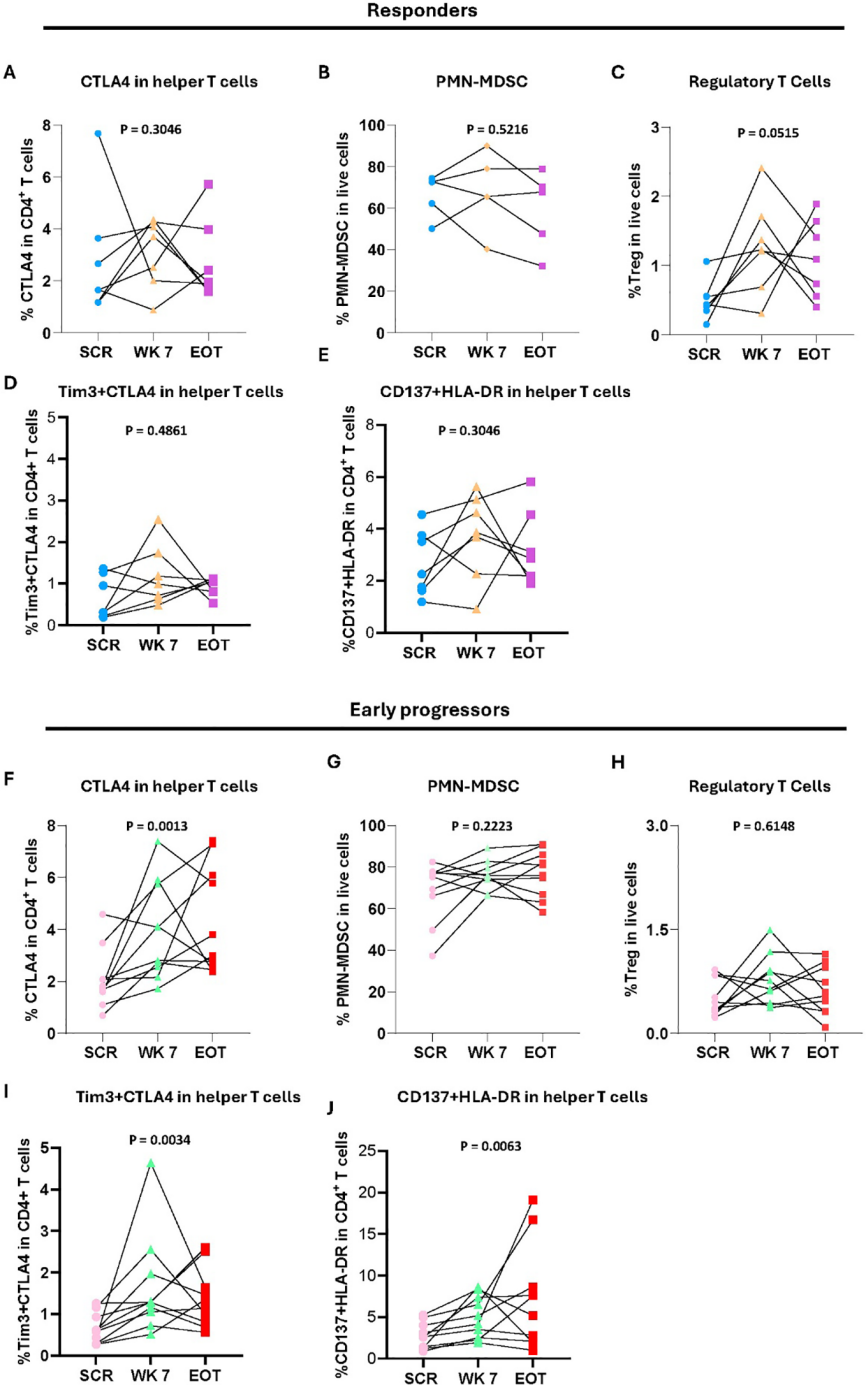
Changes in immune cell subsets in responders and early progressors during treatment: **(A-E)** Line graphs showing the percentage of **(A)** CTLA4 in helper T cells; **(B)** PMN-MDSC in live cells; **(C)** Regulatory T cells in live cells; **(D)** Tim3+CTLA4 in helper T cells; **(E)** CD137+HLA-DR in helper T cells in responders. Note: Panel B has 5 responders due to technical error; **(F-J)** Line graphs showing the percentage of **(F)** CTLA4 expression in helper T cells. **(G)** PMN-MDSC in live cells. **(H)** Regulatory T cells in live cells. **(I)** Tim3+CTLA4 in helper T cells. **(J)** CD137+HLA-DR in helper T cells in early progressors (n = 10). Significance difference was tested by Friedman Test, with multiple comparison using the Benjamini–Hochberg false discovery rate (FDR) procedure. Significance difference was accepted at p ≤ 0.05. SCR, screening. WK 7, week 7. EOT, end of treatment.

Although no statistically significant differences were observed, early progressors at<week 26 showed a trend towards higher levels of CD137^+^HLA-DR^+^CD4^+^, CD4^+^CTLA4^+^, Tim3^+^CTLA4^+^CD4^+^ T cells, as well as neutrophils/PMN-MDSCs (**Supplementary Figures 8A–D**). In contrast, responders exhibited a trend towards higher levels of Tregs at week 26 (**Supplementary Figure 8E**). To determine whether these differences were pre-existing or influenced by treatment, we analyzed paired samples collected at screening, week 7, and EOT in both early progressors and responders. In responders, while there were no significant differences observed in other cell populations (**Figures 4A, B, D, E**), Tregs exhibited a trend toward a gradual increase over time, (**Figure 4C**). In early progressors, CD4^+^CTLA-4^+^ (**Figure 4F**), Tim-3^+^CTLA-4^+^CD4^+^ and CD137^+^HLA-DR^+^CD4^+^ T cells (**Figures 4I, J**), showed a significant increase by EOT, suggesting that these changes may develop during therapy rather than being entirely pre-existing. Consistent with our analyses in responders and non-responders, we compared all ICMs between screening and week 7, as well as between screening and EOT in responders and early progressors. No clear changes were observed between screening and week 7 (data not shown). Therefore, subsequent analyses focused on screening vs. EOT comparisons. In early progressors (screening vs.<week 26), a trend towards increased e-MDSCs, CD80^+^ pDCs and kyn/tryp ratio was observed (**Supplementary Figures 8F, G, I**), along with a decrease in the total pDCs (**Supplementary Figure 8H**). In responders, trends towards decreased CD56^+^CD16^+^ NK cells between screening and week 26 were observed (**Supplementary Figure 8J**).

## Discussion

In the PRIMMO study (NCT03192059), we evaluated the clinical activity of a multimodal treatment strategy combining pembrolizumab, radiotherapy and IDC in patients with advanced CC and EC who had received at least one prior line of chemotherapy. While the study failed to achieve its primary endpoint (22.2% BORR for CC and 12.0% BORR for EC) (38), the accompanying translational immune analyses provide important insights into systemic immune dynamics that may help explain the observed clinical outcomes.

In this exploratory study, we performed longitudinal immune profiling of peripheral blood to characterize both baseline immune states and treatment-associated changes. Overall, our findings, to be considered as hypothesis-generating, suggest that immune dynamics during treatment, rather than baseline features alone, may better distinguish treatment response. Importantly, our findings suggest that effective ICB-based therapy may depend on a coordinated immune balance, rather than on isolated activation signals (45, 46).

At baseline, no single ICM clearly differentiated responders from non-responders. However, a composite immune score derived from 13-ICMs showed an association with clinical outcomes, with lower scores appearing to correlate with treatment benefit and improved survival. Although this score demonstrated discriminatory potential within this dataset, it was derived *post hoc* and lacks external validation, limiting its immediate applicability. Nevertheless, this observation supports the concept that integrated immune profiles may reflect systemic immune complexity better than individual biomarkers, consistent with observations in other malignancies (47, 48). Although integrated immune cell signatures in peripheral blood have been less studied in CC and EC, baseline composite immune scores combining lymphoid and myeloid subsets have been associated with improved survival and response to ICB across multiple malignancies (49, 50). Furthermore, a higher combined score of T-cell subsets at baseline correlated with treatment response and prolonged time to progression, highlighting their potential as predictive biomarkers for ICB efficacy (51). Beyond composite analyses, elevated levels of activated NK cell and CD4^+^ T cell phenotypes were linked to poorer survival. NK cells are innate lymphocytes important for immune surveillance and tumor control, comprising 2-31% of peripheral blood lymphocytes. CD56^+^CD16^+^ NK cells have high cytolytic potential, whereas CD56^bright^CD16^-/+^ NK cells secrete cytokines such as interferon (IFN)-gamma, TGF- beta and IL-10, to regulate adaptive immunity. CD56^-^CD16^+^ NK cells are less cytotoxic and secrete fewer cytokines (52, 53). Unlike antigen-specific T cells, NK cells can eliminate targets without any antigen or MHC restrictions, and they express immune checkpoints, including PD-(L)1, making them potential targets of ICB (54).

Despite the prognostic and predictive relevance of peripheral NK cells and their subsets, studies across different tumor types and ICB regimens have yielded inconsistent results (55). In our cohort, elevated baseline levels of CD161^+^CD56^+^CD16^+^ NK cells were associated with shorter PFS, consistent with reports in other tumor types where certain NK phenotypes may reflect impaired or dysregulated function (56, 57). For example, Seymour et al. reported that increased levels of activated CD56^+^CD16^+^ NK cells in baseline blood of multiple myeloma patients, were associated with worse OS and PFS (57).

Longitudinally, non-responders demonstrated further increases in activated NK cell subsets. Contrary to a recent study (58), non-responders experienced an increase in activated CD56^+^CD16^+^ and CD56^bright^ NK cells. While NK cells are generally regarded as anti-tumor effectors, emerging evidence suggests that, under certain conditions, they may also suppress adaptive immune responses by reducing CD8^+^ T cell activity against tumor cells, potentially limiting treatment efficacy (59, 60). Together, these findings suggest that NK cells may have context-dependent roles in shaping responses to ICB-based therapy. Monitoring changes in NK-cell subsets over time could therefore provide additional insight into treatment-related immune dynamics. T cell dynamics further supported a complex immune landscape in non-responders. In our cohorts, higher baseline levels of CD4^+^ T cells expressing CTLA-4^+^PD-1^+^ were associated with poorer OS. Early treatment dynamics further indicated significant changes in T-cell compartments in non-responders. Contrary to previous findings (49, 61), non-responders exhibited higher frequencies of activated CD4^+^ and CD8^+^ T-cell phenotypes accompanied by co-expression of inhibitory markers, suggesting potential disruption of global immune coordination (62). CD4^+^ T cells not only support CD8^+^ T-cell priming through DC licensing (61), but also enhance the tumoricidal activity of macrophages and NK cells (62, 63), emphasizing their importance in orchestrating anti-tumor immunity. While co-expression of inhibitory receptors has been associated with exhaustion-like states in chronic antigen exposure (64), we did not perform functional assays, and therefore cannot determine whether these cells are functionally impaired. Importantly, such phenotypes may also reflect ongoing activation under regulatory constraints rather than terminal dysfunction (64). Similar to the early phase, non-responders continued to show sustained activation and inhibitory phenotypic features in both CD4^+^ and CD8^+^ T cells during the late treatment phase, suggesting a state of ongoing immune perturbation rather than transient changes. Comparable patterns have been reported in other clinical settings, including lung cancer patients who received chemoradiation, where sustained peripheral T-cell activation accompanied by exhaustion markers were associated with poorer survival outcomes (65). In parallel, non-responders showed increased levels of Tregs, M-MDSCs, and kyn/tryp ratio, accompanied by reduced frequencies of CD80^+^ pDCs suggesting coordinated activation of metabolic and immunoregulatory pathways. These observations are consistent with prior studies linking high Tregs level in advanced HER2-negative breast cancer and NSCLC (66, 67) and M-MDSC levels in melanoma patients (68) to poor ICB outcome. In lung cancer patients who received first-line radiotherapy, an increase in kyn/tryp ratio was linked to worse OS and PFS (69). Furthermore, in the late phase, non-responders, including those with early progression, displayed elevated levels of PMN-MDSCs and e-MDSCs. In contrast, no statistically significant longitudinal immune changes were observed in responders after multiple testing correction. This finding should be interpreted with caution, as the small number of responders (n=7) and inter-patient variability may mask small but meaningful differences.

Tumor-type-specific analyses further suggested differences in immune dynamics, with NK cell-associated changes more prominent in CC and T cell-associated changes in EC. These observations may reflect underlying biological differences, including viral antigen-driven immunity in CC and molecular heterogeneity in EC (70, 71), although confirmation in larger cohorts is required.

We also observed increased sPD-1 levels in non-responders. The biological and clinical relevance of sPD-1 remains unclear. It has been proposed that the increase in sPD-1 after treatment may positively affect the cancer-immune cycle by increasing antigen presentation, restoring cytotoxic T-cell activity and enhancing overall anti-cancer immune response (72). However, other studies have shown that increased sPD-1 may interfere with anti-PD-1 binding to membrane bound PD-1 on immune cells, thereby potentially reducing treatment efficacy (73).

It is important to emphasize that all immune observations in this study are based on phenotypic profiling. NK cell subsets, CD4^+^ and CD8^+^ T cells, as well as Treg and MDSC subsets, were defined using established phenotypic markers without functional validation. Therefore, the observed changes should be interpreted as phenotypic associations rather than definitive evidence of functional immune states. Future studies incorporating functional assays will be essential to clarify the biological significance of these findings.

Beyond the clinical activity observed in the PRIMMO trial, our exploratory immune-profiling analyses offer preliminary insights into how systemic immune features may dynamically and context-dependently shape responses to ICB-based therapy in CC and EC. While our study was not powered to define predictive or prognostic biomarkers, the patterns observed point to immune characteristics that merit further investigation in larger, independent cohorts. In particular, the limited number of responders constrains the interpretation of longitudinal immune changes within this subgroup.

Notably, our findings suggest that non-response may not simply reflect a suppressed or absent immune state, but may instead be associated with a concurrent increase in both activating and inhibitory/regulatory immune features (74, 75).This pattern may be indicative of a state of systemic immune imbalance, where immune activation is accompanied by counter-regulatory mechanisms that could limit effective anti-tumor responses. Such observations provide a more nuanced perspective on therapeutic response, suggesting that it may not be determined solely by the magnitude of immune activation, but also by the balance between effector and regulatory pathways, which may be critical for clinical efficacy (76). Capturing this balance requires a systems-level perspective that considers the dynamic interplay between immune effector and regulatory mechanisms. Advanced computational approaches such as Bayesian dynamic modeling, network-based inference, and integrative multi-omics analysis offer tools to quantify immune balance by integrating data from diverse cellular states and soluble mediators.

In CC and EC, where blood-based immune data remain relatively limited despite the growing use of ICB-based therapies, our results support the integration of longitudinal immune monitoring into clinical trials. Current biomarker strategies are still largely based on baseline measurements, which may not fully capture the evolving nature of treatment-induced immune responses. Longitudinal assessment could therefore provide additional, clinically relevant insights by tracking immune dynamics over time. Such approaches may help to identify, in a time-dependent manner, which patients are more likely to benefit from therapy and when these responses emerge. They may also facilitate the early detection of response or resistance patterns, thereby supporting more timely and informed clinical decision-making. Importantly, blood-based immune markers represent a minimally invasive strategy and could complement established tumor-centered biomarkers, such as PD-L1 expression, MMR status, and tumor mutational burden (TMB), to improve patient stratification and guide therapeutic choices. Blood-based immune markers offer a minimally invasive approach and could complement established tumor-centered biomarkers, such as PD-L1 expression, MMR status, and TMB, to enhance patient stratification and guide therapeutic decisions (77, 78). While our study was not designed to define definitive biomarkers, the trends we observed highlight immune characteristics that merit further investigation in larger, independent cohorts.

From a broader clinical and translational perspective, this study highlights important considerations. First, systemic immune responses during ICB-based therapy appear to be dynamic and continuously evolving, highlighting the limitations of relying solely on baseline biomarkers. Second, non-response may reflect complex immune remodeling rather than a simple absence of immune activation. Third, an integrated evaluation of immune balance may provide more informative insights than single-parameter biomarkers alone. Together, these observations underscore the importance of adopting more comprehensive and longitudinal approaches to immune monitoring in future studies.

## Limitations

While this study provides valuable insights into the immunological effects of ICB-based combination therapy, a number of limitations should be acknowledged. The study cohort was small and heterogeneous, reducing statistical power and limiting generalizability. The absence of a randomized design and monotherapy control arm also prevents definitive attribution of clinical benefit to the combination regimen versus anti-PD-1 therapy alone.

Functional immune assays were not conducted, limiting mechanistic interpretation, and the lack of immunohistochemical validation due to limited tissue availability and technical constraints restricted assessment of the TME. Statistical robustness was limited by the absence of validation cohorts, lack of multivariate analyses, incomplete or inconsistent sampling, and use of arbitrary ROC-based cut-offs. The combined score was generated *post hoc* from the same dataset, and its high AUC (0.95) likely reflects overfitting, and should be regarded as an upper-bound estimate of discriminatory performance.

Despite these limitations, a key strength of this study is the incorporation of multiparametric immune profiling, encompassing soluble inflammatory mediators, lymphoid, and myeloid subsets. Importantly, our work represents one of the few efforts to characterize circulating immune profiles in advanced CC and EC patients receiving ICB-based combination therapy, with potential implications for biomarker discovery.

## Conclusion

This study provides insight into the systemic immune effects of ICB-based combination therapy in CC and EC. While clinical activity observed in the PRIMMO trial was modest, the accompanying immune analyses suggest that non-response is associated with dynamic and coordinated changes in both immune activation and regulation. These observations support the view that treatment outcomes may not be determined solely by the presence of immune activation, but rather by the broader balance between effector and regulatory immune processes. In this context, peripheral immune profiling represents a promising complementary approach to tumor-based biomarkers, offering additional opportunities to capture treatment-relevant immune changes in a minimally invasive manner. Although the present findings remain exploratory, they highlight immune patterns that warrant further investigation. Future studies should aim to validate these findings in larger, independent cohorts and to assess their reproducibility across different clinical settings. Beyond validation, there is a clear need to move beyond single timepoint assessments and incorporate longitudinal and integrative immune monitoring strategies, which may better reflect the dynamic nature of treatment-induced immune responses. Efforts to further elucidate the functional interplay between systemic and tumor-infiltrating immune populations will also be important for advancing our understanding of response mechanisms. In parallel, the development of robust preclinical and computational models capable of capturing immune dynamics may help to refine these insights and support the identification of strategies to restore immune balance within the tumor microenvironment. Ultimately, a more comprehensive understanding of systemic immune regulation and its interaction with local tumor immunity may contribute to improved patient stratification, more accurate prediction of treatment response, and the continued optimization of ICB-based therapeutic approaches in gynecologic cancers.

## Data Availability

The raw data supporting the conclusions of this article will be made available by the authors, without undue reservation.
