# Supplementary material for: Circulating immune cell profiling in advanced cervical and endometrial cancer patients treated with PD-1 blockade, radiotherapy, and immune modulation in the PRIMMO trial
Source: Front Immunol. 2026 May 21;17:1794131. doi: 10.3389/fimmu.2026.1794131 (PMC13233545; doi:10.3389/fimmu.2026.1794131)
Supplement: Supplementary file 1 [file Table1.docx]

***Supplementary tables***

| **Antibody target** | **Fluorochrome** | **Clone** | **Isotype/light chain** | **Supplier** |
| --- | --- | --- | --- | --- |
| Fixable viability dye | eFluor506 | – | – | Thermo Fisher |
| CD4 | FITC | RPA-T4 | Mouse IgG1/κ | Biolegend |
| CD127 | PerCP-Cy5.5 | HIL-7R-M21 | Mouse IgG1/κ | BD Biosciences |
| CD3 | PE/Cy7 | HIT3a | Mouse IgG2a/κ | Biolegend |
| CD8 | APC-H7 | SK1 | Mouse IgG1/κ | BD Biosciences |
| CD45 | Pacific Blue | HI30 | Mouse IgG1/κ | Biolegend |
| CD25 | PE | 2A3 | Mouse IgG1/κ | BD Biosciences |
| FoxP3 | APC | 236A/E7 | Mouse IgG1/κ | Thermo Fisher |
| HLA-DR | APC-H7 | L243 | Mouse IgG2a, κ | BD biosciences |
| Tim-3 | PE | F38-2E2 | Mouse IgG1, κ | Biolegend |
| ICOS (CD278) | PerCP-Cy5.5 | C398.4A | Armenian Hamster IgG | Biolegend |
| CTLA-4 | APC | L3D10 | Mouse IgG1, κ | Biolegend |
| PD-1 (CD279) | BV421 | EH12.2H7 | Mouse IgG1, κ | Biolegend |
| CD8 | FITC | HIT8a | Mouse IgG1, κ | Biolegend |
| CD137 | PE | 4B4-1 | Mouse IgG1, κ | Biolegend |
| CD4 | PerCP-Cy5.5 | RPA-T4 | Mouse IgG1, k | Biolegend |
| CD69 | BV421 | FN50 | Mouse IgG1, k | BD biosciences |
| CD45 | FITC | HI30 | Mouse IgG1, κ | Biolegend |
| CD161 | PE | HP-3G10 | Mouse IgG1, κ | Biolegend |
| CD16 | PerCP-Cy5.5 | 3G8 (RUO) | Mouse BALB/c x DBA/2 | BD biosciences |
| CD3 | APC | OKT3 | Mouse IgG2a, κ | Biolegend |
| CD56 | PE-Cy7 | MEM-188 | Mouse IgG2a, κ | Biolegend |
| HLA-ABC | PE | W6/32 | Mouse IgG2a, κ | Biolegend |
| CD14 | PerCP-Cy5.5 | M5E2 | Mouse IgG2a, κ | BD biosciences |
| CD123 | PE-Cy7 | 6H6 | Mouse IgG1, κ | Invitrogen |
| CD11c | Pacific Blue | Bu15 | Mouse IgG1, κ | Biolegend |
| CD80 | AF647 | 2D10 | Mouse IgG1, κ | Biolegend |
| CD45 | FITC | REA747 | recombinant human IgG1 | Miltenyi Biotec |
| CD11b | PE | REA713 | recombinant human IgG1 | Miltenyi Biotec |
| CD14 | PerCP-Vio700 | REA599 | recombinant human IgG1 | Miltenyi Biotec |
| CD19 | PE-Vio770 | REA675 | recombinant human IgG1 | Miltenyi Biotec |
| CD3 | PE-Vio770 | REA613 | recombinant human IgG1 | Miltenyi Biotec |
| CD56 | PE-Vio770 | REA196 | recombinant human IgG1 | Miltenyi Biotec |
| CD15 | APC | VIMC6 | mouse IgM | Miltenyi Biotec |
| HLA-DR | APC-Vio770 | REA805 | recombinant human IgG1 | Miltenyi Biotec |
| CD33 | VioBlue | REA775 | recombinant human IgG1 | Miltenyi Biotec |
| Anti-h/m Arginase | FITC | Met1-Lys322 | Sheep IgG | R&D Systems |

**Supplementary Table 1. List and details of fluorescently conjugated anti-human monoclonal antibodies**

APC, allophycocyanin; CD, cluster of differentiation, FITC, fluorescein isothiocyanate; PE, phycoerythrin; HLA-DR, & ABC, human leukocyte antigen –DR and ABC isotype; AF647, Alexa Fluor 647 dye; PD-L1, programmed death ligand-1.

**Supplementary Table 2: Different immune cell types and their immunomodulatory profile**

| **Cell/Marker** | **Cell type** | **Immunomodulatory profile** | **Cell/Marker** | **Cell type** | **Immunomodulatory profile** |
| --- | --- | --- | --- | --- | --- |
| CD3^+^ | Total T cells | Activation | CD8^+^PD1^+^ | Cytotoxic T cells | Inhibitory |
| CD4^+^ | Helper T cells | Activation | CD8^+^Tim3^+^ | Cytotoxic T cells | Inhibitory |
| CD4^+^CD69^+^ | Helper T cells | Activation | CD8^+^Tim3^+^CTLA4^+^ | Cytotoxic T cells | Inhibitory |
| CD4^+^CD137^+^ | Helper T cells | Activation | CD8^+^Tim3^+^ICOS^+^ | Cytotoxic T cells | Inhibitory |
| CD4^+^HLA-DR^+^ | Helper T cells | Activation | CD8^+^Tim3^+^PD1^+^ | Cytotoxic T cells | Inhibitory |
| CD4^+^CD69^+^HLA-DR^+^ | Helper T cells | Activation | CD8^+^CTLA4^+^ICOS^+^ | Cytotoxic T cells | Inhibitory |
| CD4^+^CD69^+^CD137^+^ | Helper T cells | Activation | CD8^+^CTLA4^+^PD-1^+^ | Cytotoxic T cells | Inhibitory |
| CD4^+^CD137^+^HLA-DR^+^ | Helper T cells | Activation | CD8^+^ICOS^+^PD-1^+^ | Cytotoxic T cells | Inhibitory |
| CD4^+^CTLA4^+^ | Helper T cells | Inhibitory | CD4^+^CD25^+^FOXP3^+^ | Regulatory T cells | Inhibitory |
| CD4^+^ICOS^+^ | Helper T cells | Activation | CD56^+^CD16^+^ | CD56^+^CD16^+^ NK cells | Activation |
| CD4^+^PD1^+^ | Helper T cells | Inhibitory | CD56^+^CD16^+^CD69^+^ | CD56^+^CD16^+^CD69^+^ NK cells | Activation |
| CD4^+^Tim3^+^ | Helper T cells | Inhibitory | CD56^+^CD16^+^CD161^+^ | CD56^+^CD16^+^CD161^+^ NK cells | Activation |
| CD4^+^Tim3^+^ICOS^+^ | Helper T cells | Inhibitory | CD56^+^CD16^+^HLA-DR^+^ | CD56^+^CD16^+^HLA-DR^+^NK cells | Activation |
| CD4^+^CTLA4^+^ICOS^+^ | Helper T cells | Inhibitory | CD56^-^CD16^+^ | CD56^-^CD16^+^ NK cells | Inhibitory |
| CD4^+^CTLA4^+^PD1^+^ | Helper T cells | Inhibitory | CD56^-^CD16^+^ CD69^+^ | CD56^-^CD16^+^ CD69^+^ NK cells | Inhibitory |
| CD8^+^ | Cytotoxic T cells | Activation | CD56^-^CD16^+^ CD161^+^ | CD56^-^CD16^+^ CD161+ NK cells | Inhibitory |
| CD8^+^CD69^+^ | Cytotoxic T cells | Activation | CD56^-^CD16^+^ HLA-DR^+^ | CD56^-^CD16^+^ HLA-DR^+^ NK cells | Inhibitory |
| CD8^+^CD137^+^ | Cytotoxic T cells | Activation | CD56^bright^ | CD56^bright^ NK cells | Activation |
| CD8^+^HLA-DR^+^ | Cytotoxic T cells | Activation | CD56^bright+^CD69^+^ | CD56^bright+^CD69^+^NK cells | Activation |
| CD8^+^CD69^+^CD137^+^ | Cytotoxic T cells | Activation | CD56^bright+^CD161^+^ | CD56^bright+^CD161^+^NK cells | Activation |
| CD8^+^CD137^+^HLA-DR^+^ | Cytotoxic T cells | Activation | CD56b^right+^HLA-DR^+^ | CD56^bright+^HLA-DR^+^is iNK cells | Activation |
| CD8^+^CTLA4^+^ | Cytotoxic T cells | Inhibitory | CD11c^+^CD123^-^ | Myeloid DC | Activation |
| CD8^+^ICOS^+^ | Cytotoxic T cells | Activation | CD11c^+^CD123^-^ CD80^+^ | Myeloid DC | Activation |
| Arginase-1 activity | Arginase | Inhibitory | CD11c^+^CD123^-^ HLA-ABC^+^ | Myeloid DC | Activation |
| sPD-1 | Soluble PD-1 | Inhibitory | CD11c^-^CD123^+^ | Plasmacytoid DC | Activation |
| sPD-L1/2 | Soluble PD-L1/2 | Inhibitory | CD11c^-^CD123^+^CD80^+^ | Plasmacytoid DC | Activation |
| sPD-L2 | Soluble PD-L2 | Inhibitory | CD11c-CD123^+^HLA-ABC^+^ | Plasmacytoid DC | Activation |
| Kyn/tryp ratio | Kynurenine-tryptophan | Inhibitory | CD45^+^Lin-CD14-HLA-DRlo-CD11b^+^CD33^+^ | Early-stage MDSC | Inhibitory |
| CD45+ CD14+ HLA-DRlowCD11b+ CD15+ | Mononuclear MDSC | Inhibitory | CD45^+^CD14^-^CD15^+^ CD11b^+^ | Polymorphonuclear MDSC | Inhibitory |

CD, cluster of differentiation; HLA-DR, human leukocyte antigen –DR isotype; MDSC, myeloid-derived suppressor cell; DC, dendritic cell; NK, natural killer cell; CTLA4, Cytotoxic T-Lymphocyte Associated Protein 4; ICOS, Inducible T Cell Co-stimulator; PD-1, Programmed Death Receptor 1; PD-L1, programmed death ligand-1; Tim3, T cell immunoglobulin and mucin domain-containing protein 3

**Supplementary Table 3: Subgroup analysis and total number of samples collected at different time points**

| **Time point** | **Subgroup** | |
| --- | --- | --- |
| **Tumor type** | | |
|  | **Cervical (N)** | **Endometrial (N)** |
| Screening | 19 | 24 |
| Week 7 | 16 | 20 |
| End of treatment (EOT) | 11 | 12 |
| Screening vs. Week 7 | 16 | 20 |
| Screening vs. EOT | 11 | 12 |
| **Analysis in responders and non-responders** | | |
|  | **Responders (N)** | **Non-responders (N)** |
| Screening | 7 | 36 |
| Week 7 | 7 | 29 |
| EOT | 7 | 16 |
| Screening vs. Week 7 | 7 | 29 |
| Screening vs. EOT | 7 | 16 |
| **Analysis in responders and early progressors** | | |
|  | **Responders (N)** | **Early progressors (N)** |
| Screening | 7 | 13 |
| Week 7 | 7 | 12 |
| EOT | 7 | 13 |
| Screening vs. Week 7 | 7 | 12 |
| Screening vs. EOT | 7 | 13 |

EOT; end of treatment, N; total number of samples

**Supplementary Table 4: Patient characteristics and treatment outcome**

| **Patient ID** | **Tumor types** | **Histology** | **Grade** | **Prior systemic treatment** | **Disease status** | **BOR by RECIST** | **Response category** | **HPV status** | **Molecular subtype** |
| --- | --- | --- | --- | --- | --- | --- | --- | --- | --- |
| PRI-PT-01 | Cervical | Squamous | G3 | 1 | Refractory | PD | NR | Positive |  |
| PRI-PT-02 | Cervical | Adenosquamous | G2 | 2 | Recurrent | PD | NR | Negative | - |
| PRI-PT-03 | Endometrial | Endometrioid | G1 | 4 | Recurrent/Refractory | SD | NR | - | MSI-H |
| PRI-PT-04 | Cervical | Adenosquamous | G3 | 2 | Recurrent/Refractory | PD | NR | Positive | - |
| PRI-PT-05 | Endometrial | Endometrioid | G3 | 2 | Recurrent/Refractory | SD | NR | - | MSI-H |
| PRI-PT-06 | Cervical | Squamous | G3 | 1 | Recurrent | PD | NR | Positive | - |
| PRI-PT-09 | Endometrial | Endometrioid | G1 | 2 | Recurrent/Refractory | PD | NR | - |  |
| PRI-PT-11 | Cervical | Squamous | G3 | 2 | Refractory | PD | NR | Positive | - |
| PRI-PT-12 | Endometrial | Endometrioid | G3 | 1 | Recurrent/Refractory | SD | NR | - | MSI-H |
| PRI-PT-13 | Cervical | Squamous | G3 | 3 | Recurrent/Refractory | SD | NR | Positive | - |
| PRI-PT-14 | Cervical | Adenosquamous | G2 | 4 | Recurrent/Refractory | PD | NR | Positive | - |
| PRI-PT-15 | Cervical | Squamous | G2 | 2 | Recurrent/Refractory | CR | R | Positive | - |
| PRI-PT-16 | Endometrial | Serous | G3 | 1 | Recurrent | SD | NR | - | NOS |
| PRI-PT-17 | Cervical | Adenosquamous | G1 | 4 | Recurrent/Refractory | PD | NR | NE | - |
| PRI-PT-18 | Cervical | Adenosquamous | G3 | 1 | Recurrent | PD | NR | Positive | - |
| PRI-PT-20 | Cervical | Squamous | G3 | 2 | Recurrent | PD | NR | Positive | - |
| PRI-PT-21 | Cervical | Squamous | G3 | 2 | Refractory | PD | NR | Negative | - |
| PRI-PT-23 | Cervical | Squamous | G3 | 1 | Recurrent | PD | NR | Positive | - |
| PRI-PT-24 | Endometrial | Endometrioid | G3 | 1 | Recurrent/Refractory | PD | NR | - | NOS |
| PRI-PT-27 | Cervical | Squamous | G3 | 1 | Refractory | PD | NR | Positive | - |
| PRI-PT-28 | Endometrial | Endometrioid | G1 | 3 | Recurrent | SD | NR | - | NSMP |
| PRI-PT-29 | Cervical | Squamous | G3 | 2 | Recurrent | PR | R | Positive | - |
| PRI-PT-32 | Endometrial | Endometrioid | G3 | 1 | Recurrent/Refractory | PD | NR | - | p53abn |
| PRI-PT-34 | Endometrial | Endometrioid | G1 | 5 | Recurrent/Refractory | PD | NR | - | MSI-H |
| PRI-PT-37 | Endometrial | Endometrioid | G2 | 1 | Recurrent | PR | R | - | MSI-H |
| PRI-PT-38 | Cervical | Squamous | G3 | 1 | Recurrent | PR | R | Positive | - |
| PRI-PT-41 | Endometrial | Serous | G3 | 1 | Refractory | PD | NR | - | p53abn |
| PRI-PT-42 | Endometrial | Serous | G3 | 1 | Refractory | PD | NR | - | p53abn |
| PRI-PT-43 | Endometrial | Serous | G3 | 4 | Recurrent/Refractory | PD | NR | - | NSMP |
| PRI-PT-45 | Endometrial | Serous | G3 | 2 | Recurrent/Refractory | PD | NR | - | p53abn |
| PRI-PT-46 | Endometrial | Endometrioid | G3 | 3 | Refractory | PD | NR | - | NSMP |
| PRI-PT-47 | Cervical | Adenosquamous | G2 | 1 | Recurrent/Refractory | PR | R | Positive | - |
| PRI-PT-50 | Endometrial | Endometrioid | G2 | 1 | Recurrent | PD | NR | - | MSI-H |
| PRI-PT-51 | Endometrial | Serous | G3 | 2 | Recurrent/Refractory | PR | R | - | p53abn |
| PRI-PT-52 | Endometrial | Serous | G3 | 1 | Refractory | PR | R | - | p53abn |
| PRI-PT-53 | Endometrial | Serous | G3 | 1 | Recurrent | PD | NR | - | p53abn |
| PRI-PT-54 | Endometrial | Serous | G3 | 1 | Recurrent/Refractory | PD | NR | - | NOS |
| PRI-PT-56 | Cervical | Squamous | G3 | 1 | Refractory | PD | NR | Positive | - |
| PRI-PT-58 | Endometrial | Endometrioid | G3 | 2 | Refractory | SD | NR | - | NOS |
| PRI-PT-60 | Cervical | Squamous | G3 | 3 | Recurrent/Refractory | PD | NR | Positive | - |
| PRI-PT-61 | Endometrial | Endometrioid | G3 | 1 | Recurrent/Refractory | PD | NR | - | MSI-H |
| PRI-PT-62 | Endometrial | Serous | G3 | 1 | Refractory | PD | NR | - | p53abn |
| PRI-PT-63 | Endometrial | Serous | G3 | 1 | Recurrent | PD | NR | - | p53abn |

CR, complete response; PR, partial response; SD, stable disease; PD progressive disease; NOS, not otherwise specified; NE, not evaluable; HPV, human papillomavirus; MSI, microsatellite instability; NSMP, non-specific molecular profile.

**Supplementary Table 5: Mean, Median, and P-values of cell marker expression: cervical vs. endometrial cancer at screening**

| **Cell/Marker** | **Cervical Cancer** | | **Endometrial Cancer** | | **P-value** |
| --- | --- | --- | --- | --- | --- |
|  | **Mean** | **Median** | **Mean** | **Median** |  |
| CD3^+^ | 28.33 | 26.70 | 30.63 | 27.65 | 0.9390 |
| CD4^+^ | 15.06 | 14.40 | 16.64 | 18.60 | 1. 0000 |
| CD4^+^CD69^+^ | 3.559 | 3.320 | 3.580 | 3.155 | 1. 0000 |
| CD4^+^CD137^+^ | 2.866 | 2.350 | 3.071 | 2.880 | 1. 0000 |
| CD4^+^HLA-DR^+^ | 5.554 | 4.670 | 5.655 | 5.035 | 0.9408 |
| CD4^+^CD69^+^HLA-DR^+^ | 2.727 | 2.190 | 3.462 | 2.890 | 1. 0000 |
| CD4^+^CD69^+^CD137^+^ | 1.248 | 0.9400 | 1.057 | 0.8350 | 0.9361 |
| CD4^+^CD137^+^HLA-DR^+^ | 2.873 | 2.580 | 3.346 | 2.900 | 1. 0000 |
| CD4^+^CTLA4^+^ | 2.392 | 2.080 | 2.463 | 1.945 | 0.9355 |
| CD4^+^ICOS^+^ | 8.867 | 8.350 | 8.867 | 8.753 | 0.9987 |
| CD4^+^PD1^+^ | 5.646 | 4.350 | 4.445 | 3.700 | 1. 0000 |
| CD4^+^Tim3^+^ | 4.358 | 2.810 | 4.786 | 2.845 | 0.9447 |
| CD4^+^Tim3^+^ICOS^+^ | 5.786 | 5.320 | 4.916 | 4.880 | 1. 0000 |
| CD4^+^CTLA4^+^ICOS^+^ | 1.089 | 1.000 | 1.295 | 1.175 | 1. 0000 |
| CD4^+^CTLA4^+^PD1^+^ | 2.499 | 1.570 | 1.780 | 1.575 | 0.9228 |
| CD8^+^ | 10.72 | 10.30 | 12.52 | 11.95 | 0.9988 |
| CD8^+^CD69^+^ | 7.074 | 7.020 | 9.360 | 6.720 | 1. 0000 |
| CD8^+^CD137^+^ | 3.902 | 3.750 | 4.705 | 3.975 | 1. 0000 |
| CD8^+^HLA-DR^+^ | 6.421 | 5.150 | 6.570 | 5.565 | 0.9403 |
| CD8^+^CD69^+^CD137^+^ | 5.299 | 4.590 | 6.130 | 3.090 | 1. 0000 |
| CD8^+^CD137^+^HLA-DR^+^ | 4.001 | 4.140 | 4.413 | 3.440 | 0.9321 |
| CD8^+^PD1^+^ | 3.777 | 3.020 | 3.659 | 2.840 | 0.9791 |
| CD8^+^Tim3^+^ | 2.944 | 2.790 | 3.095 | 1.935 | 0.9103 |
| CD8^+^CTLA4^+^ | 3.712 | 2.510 | 5.440 | 2.490 | 1. 0000 |
| CD8^+^ICOS^+^ | 6.429 | 4.450 | 6.427 | 6.305 | 0.9194 |
| CD8^+^Tim3^+^CTLA4^+^ | 1.158 | 0.7400 | 1.794 | 0.7600 | 1. 0000 |
| CD8^+^Tim3^+^ICOS^+^ | 2.268 | 1.830 | 1.945 | 1.900 | 0.9700 |
| CD8^+^Tim3^+^PD1^+^ | 2.012 | 1.300 | 1.792 | 1.395 | 0.9775 |
| CD8^+^CTLA4^+^ICOS^+^ | 1.332 | 1.120 | 2.402 | 1.225 | 1. 0000 |
| CD8^+^CTLA4^+^PD-1^+^ | 2.398 | 2.290 | 1.897 | 1.660 | 1. 0000 |
| CD8^+^ICOS^+^PD-1^+^ | 2.463 | 2.260 | 2.319 | 2.090 | 1. 0000 |
| CD4^+^CD25^+^FOXP3^+^ | 3.360 | 3.090 | 4.256 | 3.845 | 1. 0000 |
| CD56^+^CD16^+^ | 7.333 | 6.290 | 7.798 | 7.250 | 1. 0000 |
| CD56^+^CD16^+^CD69^+^ | 1.458 | 1.240 | 4.064 | 1.635 | 1. 0000 |
| CD56^+^CD16^+^CD161^+^ | 3.183 | 2.300 | 4.000 | 2.065 | 0.9434 |
| CD56^+^CD16^+^HLA-DR^+^ | 14.06 | 13.10 | 13.69 | 12.25 | 0.9598 |
| CD56^-^CD16^+^ | 3.502 | 3.450 | 4.254 | 3.570 | 0.9978 |
| CD56^-^CD16^+^ CD69^+^ | 1.721 | 1.420 | 3.461 | 1.720 | 1. 0000 |
| CD56^-^CD16^+^ CD161^+^ | 8.098 | 4.575 | 5.903 | 4.390 | 0.9938 |
| CD56^-^CD16^+^ HLA-DR^+^ | 41.86 | 45.20 | 46.18 | 47.85 | 0.9970 |
| CD56^bright^ | 1.335 | 0.9900 | 1.292 | 1.040 | 0.9004 |
| CD56^bright+^CD69^+^ | 3.818 | 3.310 | 10.87 | 4.655 | 1. 0000 |
| CD56^bright+^CD161^+^ | 9.814 | 9.710 | 11.25 | 11.05 | 1. 0000 |
| CD56^bright+^HLA-DR^+^ | 55.52 | 62.10 | 52.78 | 51.20 | 1. 0000 |
| CD11c^+^CD123^-^ | 3.265 | 2.490 | 3.879 | 3.975 | 1. 0000 |
| CD11c^+^CD123^-^ CD80^+^ | 4.092 | 4.050 | 3.803 | 3.470 | 1. 0000 |
| CD11c^-^CD123^+^ | 0.4332 | 0.3100 | 0.4195 | 0.3450 | 0.9528 |
| CD11c^-^CD123^+^ CD80^+^ | 7.737 | 7.495 | 8.403 | 6.110 | 0.9952 |
| CD45^+^ Lin-CD14^-^ HLA-DRlo- CD11b^+^ CD33^+^ | 0.1914 | 0.1809 | 0.3058 | 0.2772 | 1. 0000 |
| CD45^+^ CD14^+^ HLA-DRlowCD11b^+^ CD15^+^ | 1.045 | 0.7960 | 1.067 | 0.9930 | 0.9267 |
| CD45^+^ CD14^-^ CD15^+^ CD11b^+^ | 66.65 | 65.40 | 67.05 | 67.00 | 0.9371 |
| Arginase-1 activity | 3.003 | 2.610 | 2.431 | 2.048 | 0.9905 |
| Kyn/tryp ratio | 94.74 | 57.65 | 90.21 | 79.92 | 1. 0000 |
| sPD-1 | 8.482 | 5.285 | 15.80 | 5.805 | 1. 0000 |
| sPD-L1 | 18.62 | 10.04 | 21.42 | 14.58 | 1. 0000 |
| sPD-L2 | 8293 | 7163 | 9088 | 8054 | 0.9270 |

CD, cluster of differentiation; HLA-DR, human leukocyte antigen –DR isotype; CTLA4, Cytotoxic T-Lymphocyte Associated Protein 4; ICOS, Inducible T Cell Co-stimulator; PD-1, Programmed Death Receptor 1; PD-L1, programmed death ligand-1; Tim3, T cell immunoglobulin and mucin domain-containing protein 3.

**Supplementary Table 6: List of immune cell markers and corresponding AUC values**

| **Cell/Marker** | **Cell type** | **AUC**  **( 95% CI)** | **Cell/Marker** | **Cell type** | **AUC (95% CI)** |
| --- | --- | --- | --- | --- | --- |
| CD11c^+^CD123^-^ | Myeloid DC | **0.351 (0.171-0.530)** | CD4^+^CD69^+^HLA-DR^+^ | Helper T cells | 0.517 (0.337-0.698) |
| CD56^bright^ | CD56^bright^ NK cells | **0.362 (0.176-0.547)** | CD8^+^Tim3^+^CTLA4^+^ | Cytotoxic T cells | 0.526 (0.341-0.710) |
| CD56^+^CD16^+^ | CD56^+^CD16^+^ NK cells | **0.375 (0.191-0.559)** | CD8^+^ICOS^+^PD-1^+^ | Cytotoxic T cells | 0.526 (0.335-0.716) |
| CD8^+^CTLA4^+^ICOS^+^ | Cytotoxic T cells | **0.386 (0.192-0.579)** | CD4^+^HLA-DR^+^ | Helper T cells | 0.530 (0.327-0.732) |
| CD4^+^ | Helper T cells | 0.410 (0.217-0.603) | CD8^+^PD1^+^ | Cytotoxic T cells | 0.532 (0.340-0.724) |
| CD8^+^Tim3^+^ | Cytotoxic T cells | 0.410 (0.246-0.574) | CD4^+^CTLA4^+^ | Helper T cells | 0.534 (0.325-0.742) |
| CD45^+^ CD14^+^ HLA^-^DRlowCD11b^+^ CD15^+^ | Mononuclear MDSC | 0.423 (0.195-0.650) | CD8^+^Tim3^+^PD1^+^ | Cytotoxic T cells | 0.534 (0.332-0.735) |
| CD11c^+^CD123^-^ CD80^+^ | Myeloid DC | 0.426 (0.222-0.630) | CD8^+^CD69^+^ | Cytotoxic T cells | 0.535 (0.348-0.722) |
| CD56^-^CD16^+^ | CD56^-^CD16^+^ NK cells | 0.438 (0.252-0.625) | CD8^+^HLA-DR^+^ | Cytotoxic T cells | 0.538 (0.349-0.726) |
| CD11c^-^CD123^+^ | Plasmacytoid DC | 0.442 (0.249-0.635) | CD8^+^ICOS^+^ | Cytotoxic T cells | 0.543 (0.361-0.725) |
| CD4^+^CD25^+^FOXP3^+^ | Regulatory T cells | 0.444 (0.256-0.631) | CD8^+^CD137^+^ | Cytotoxic T cells | 0.546 )0.357-0.735) |
| CD4^+^CD137^+^ | Helper T cells | 0.446 (0.250-0.643) | Arginase-1 activity | Arginase | 0.546 (0.351-0.740) |
| sPD-1 | Soluble PD-1 | 0.457 (0.278-0.636) | CD4^+^CD137^+^HLA-DR^+^ | Helper T cells | 0.550 (0.369-0.731) |
| CD11c^-^CD123^+^ CD80^+^ | Plasmacytoid DC | 0.461 (0.270-0.652) | CD4^+^CD69^+^ | Helper T cells | 0.559 (0.375-0.743) |
| CD56^-^CD16^+^ CD161^+^ | CD56^-^CD16^+^ CD161^+^NK cells | 0.468 (0.277-0.659) | CD4^+^Tim3^+^ICOS^+^ | Helper T cells | 0.559 (0.376-0.743) |
| CD8^+^CTLA4^+^PD-1^+^ | Cytotoxic T cells | 0.478 (0.292-0.665) | Kyn/tryp ratio | Kynurenine-tryptophan | 0.559 (0.382-0.736) |
| CD8^+^CD69^+^CD137^+^ | Cytotoxic T cells | 0.484 (0.311-0.656) | CD56^bright+^CD161^+^ | CD56^bright+^CD161^+^ NK cells | 0.560 (0.364-0.757) |
| CD11c^+^CD123^-^ HLA-ABC^+^ | Myeloid DC | 0.492 (0.299-0.685) | sPD-L1 | Soluble PD-L1/2 | 0.562 (0.390-0.734) |
| CD8^+^ | Cytotoxic T cells | 0.501 (0.318-0.684) | CD8^+^Tim3^+^ICOS^+^ | Cytotoxic T cells | 0.574 (0.390-0.758) |
| CD8^+^CTLA4^+^ | Cytotoxic T cells | 0.508 (0.320-0.696) | CD56^+^CD16^+^CD69^+^ | CD56^+^CD16^+^CD69^+^ NK cells | 0.575 (0.409-0.741) |
| CD3^+^ | Total T cells | 0.515 (0.322-0.708) | CD4^+^Tim3^+^ | Helper T cells | 0.578 (0.404-0.752) |
| CD56^+^CD16^+^HLA-DR^+^ | CD56^+^CD16^+^HLA-DR^+^ NK cells | 0.515 (0.339-0.690) | CD4^+^ICOS^+^ | Helper T cells | 0.579 (0.369-0.790) |
| CD4^+^CTLA4^+^ICOS^+^ | Helper T cells | 0.590 (0.400-0.780) | CD4^+^CD69^+^CD137^+^ | Helper T cells | **0.618 (0.448-0.789)** |
| CD45^+^ CD14^-^ CD15^+^ CD11b^+^ | Polymorphonuclear MDSC | 0.565 (0.372-0.759) | CD8^+^CD69^+^HLA-DR+ | Cytotoxic T cells | **0.621 (0.433-0.809)** |
| CD56^-^CD16^+^ CD69^+^ | CD56^-^CD16^+^ CD69^+^ NK cells | 0.593 (0.414-0.771) | CD4^+^CTLA4^+^PD1^+^ | Helper T cells | **0.664 (0.492-0.836)** |
| CD11c^-^CD123^+^ HLA-ABC^+^ | Plasmacytoid DC | 0.593 (0.392-0.794) | CD56^+^CD16^+^CD161^+^ | CD56^+^CD16^+^CD161^+^NK cells | **0.673 (0.517-0.830)** |
| CD8^+^CD137^+^HLA-DR^+^ | Cytotoxic T cells | 0.594 (0.414-0.774) | CD45^+^ Lin-CD14^-^ HLA-DRlo- CD11b^+^ CD33^+^ | Early-stage MDSC | **0.676 (0.495-0.857)** |
| CD56^-^CD16^+^ HLA-DR^+^ | CD56^-^CD16^+^ HLA-DR^+^ NK cells | **0.609 (0.409-0.809)** | sPD-L2 | Soluble PD-L2 | **0.699 (0.537-0.860)** |
| CD4^+^PD1^+^ | Helper T cells | **0.610 (0.416-0.804)** | CD56^bright+^CD69^+^ | CD56^bright+^CD69^+^ NK cells | **0.763 (0.620-0.906)** |

CD. cluster of differentiation; HLA-DR. human leukocyte antigen –DR isotype; MDSC. myeloid-derived suppressor cell; DC. dendritic cell; NK. natural killer cell; CTLA4. Cytotoxic T-Lymphocyte Associated Protein 4; ICOS. Inducible T Cell Co-stimulator; PD-1. Programmed Death Receptor 1; PD-L1. programmed death ligand-1; Tim3. T cell immunoglobulin and mucin domain-containing protein 3

**Supplementary Table 7: Univariate Cox Regression results for OS and PFS for immune cell markers**

| **Cell/Marker** | **OS** | | | **PFS** | | |
| --- | --- | --- | --- | --- | --- | --- |
|  | **HR** | **CI (95%)** | **P-value** | **HR** | **CI (95%)** | **P-value** |
| CD3^+^ | 0.993 | 0.967-1.020 | 0.621 | 0.982 | 0.957-1.007 | 0.156 |
| CD4^+^ | 0.970 | 0.925-0.017 | 0.207 | 0.956 | 0.913-1.001 | 0.055 |
| CD4^+^CD69^+^ | 1.160 | 0.995-1.351 | 0.058 | 1.257 | 1.073-1.473 | **0.005** |
| CD4^+^CD137^+^ | 1.107 | 0.865-1.417 | 0.417 | 1.120 | 0.888-1.411 | 0.339 |
| CD4^+^HLA-DR^+^ | 0.962 | 0.858-1.078 | 0.506 | 1.000 | 0.910-1.099 | 0.996 |
| CD4^+^CD69^+^HLA-DR^+^ | 1.106 | 0.875-1.398 | 0.398 | 1.171 | 0.930-1.475 | 0.178 |
| CD4^+^CD69^+^CD137^+^ | 1.735 | 1.211-2.487 | **0.003** | 2.264 | 1.51-3.326 | **<0.001** |
| CD4^+^CD137^+^HLA-DR^+^ | 1.183 | 0.914-1.532 | 0.201 | 1.250 | 0.972-1.608 | 0.083 |
| CD4^+^CTLA4^+^ | 1.038 | 0.825-1.308 | 0.749 | 0.983 | 0.789-1.224 | 0.875 |
| CD4^+^ICOS^+^ | 1.146 | 0.990-1.328 | 0.068 | 1.128 | 0.986-1.289 | 0.079 |
| CD4^+^PD-1^+^ | 1.128 | 0.983-1.295 | 0.087 | 1.101 | 0.967-1.252 | 0.145 |
| CD4^+^Tim3^+^ | 1.060 | 0.981-1.145 | 0.141 | 1.048 | 0.972-1.129 | 0.221 |
| CD4^+^Tim3^+^ICOS^+^ | 1.150 | 0.983-1.345 | 0.081 | 1.191 | 1.031-1.375 | **0.018** |
| CD4^+^CTLA4^+^ICOS^+^ | 1.366 | 0.876-2.129 | 0.169 | 1.184 | 0.821-1.707 | 0.366 |
| CD4^+^CTLA4^+^PD-1^+^ | 1.584 | 1.240-2.024 | **<0.001** | 1.428 | 1.139-1.791 | **0.002** |
| CD8^+^ | 1.007 | 0.961-1.055 | 0.780 | 0.993 | 0.953-1.035 | 0.747 |
| CD8^+^CD69^+^ | 1.020 | 0.971-1.073 | 0.431 | 1.026 | 0.982-1.071 | 0.247 |
| CD8^+^CD137^+^ | 0.987 | 0.855-1.139 | 0.858 | 0.997 | 0.875-1.137 | 0.997 |
| CD8^+^HLA-DR^+^ | 1.019 | 0.936-1.111 | 0.659 | 1.064 | 0.978-1.158 | 0.149 |
| CD8^+^CD69^+^ HLA-DR^+^ | 1.101 | 0.953-1.272 | 0.191 | 1.096 | 0.968-1.240 | 0.147 |
| CD8^+^CD69^+^CD137^+^ | 1.015 | 0.971-1.061 | 0.521 | 1.017 | 0.978-1.058 | 0.387 |
| CD8^+^CD137^+^HLA-DR^+^ | 1.093 | 0.948-1.260 | 0.219 | 1.093 | 0.963-1.240 | 0.168 |
| CD8^+^Tim3^+^ | 1.028 | 0.906-1.166 | 0.671 | 1.050 | 0.935-1.180 | 0.411 |
| CD8^+^CTLA4^+^ | 1.006 | 0.933-1.086 | 0.868 | 0.998 | 0.928-1.075 | 0.967 |
| CD8^+^ICOS^+^ | 1.024 | 0.942-1.113 | 0.575 | 1.063 | 0.977-1.155 | 0.154 |
| CD8^+^PD-1^+^ | 1.025 | 0.885-1.189 | 0.739 | 1.052 | 0.916-1.208 | 0.474 |
| CD8^+^Tim3^+^CTLA4^+^ | 1.051 | 0.880-1.257 | 0.582 | 1.005 | 0.848-1.191 | 0.954 |
| CD8+Tim3+ICOS+ | 1.137 | 0.857-1.507 | 0.374 | 1.224 | 0.940-1.593 | 0.133 |
| CD8^+^CTLA4^+^ICOS^+^ | 0.942 | 0.764-1.162 | 0.576 | 0.932 | 0.775-1.121 | 0.452 |
| CD8^+^CTLA4^+^ PD-1^+^ | 1.031 | 0.781-1.363 | 0.828 | 1.116 | 0.870-1.433 | 0.388 |
| CD8^+^ICOS^+^PD-1^+^ | 1.010 | 0.797-1.279 | 0.937 | 1.046 | 0.847-1.292 | 0.676 |
| CD8^+^Tim3^+^PD-1^+^ | 1.103 | 0.855-1.424 | 0.451 | 1.119 | 0.899-1.393 | 0.314 |
| CD4^+^CD25^+^FOXP3^+^ | 0.789 | 0.247-2.514 | 0.688 | 0.719 | 0.256-2.020 | 0.531 |
| CD56^+^CD16^+^ | 0.967 | 0.884-1.059 | 0.471 | 0.975 | 0.902-1.054 | 0.529 |
| CD56^+^CD16^+^CD69^+^ | 1.134 | 1.054-1.221 | **0.001** | 1.095 | 1.032-1.163 | **0.003** |
| CD56^+^CD16^+^CD161^+^ | 1.112 | 1.038-1.191 | **0.002** | 1.094 | 1.024-1.170 | **0.008** |
| CD56^+^CD16^+^HLA-DR^+^ | 1.011 | 0.957-1.068 | 0.687 | 1.029 | 0.976-1.084 | 0.289 |
| CD56^-^CD16^+^ | 1.002 | 0.870-1.154 | 0.983 | 1.005 | 0.888-1.137 | 0.940 |
| CD56^-^CD16^+^ CD69^+^ | 1.203 | 1.085-1.333 | **<0.001** | 1.129 | 1.034-1.232 | **0.007** |
| CD56^-^CD16^+^ CD161^+^ | 1.020 | 0.982-1.059 | 0.306 | 0.994 | 0.958-1.032 | 0.761 |
| CD56^-^CD16^+^ HLA-DR^+^ | 1.003 | 0.988-1.019 | 0.686 | 1.010 | 0.996-1.025 | 0.169 |
| CD56^bright^ | 0.855 | 0.563-1.300 | 0.465 | 0.735 | 0.455-1.187 | 0.209 |
| CD56^bright+^CD69^+^ | 1.038 | 1.015-1.061 | **0.001** | 1.026 | 1.007-1.045 | **0.006** |
| CD56^bright+^CD161^+^ | 1.057 | 0.963-1.160 | 0.243 | 1.017 | 0.927-1.115 | 0.721 |
| CD56^bright+^HLA-DR^+^ | 1.016 | 0.994-1.038 | 0.150 | 1.005 | 0.987-1.023 | 0.581 |
| CD11c^+^CD123^-^ | 0.875 | 0.714-1.072 | 0.198 | 0.955 | 0.793-1.150 | 0.626 |
| CD11c^+^CD123^-^ CD80^+^ | 0.924 | 0.753-1.134 | 0.449 | 0.941 | 0.782-1.134 | 0.525 |
| CD11c^-^CD123^+^ | 0.794 | 0.257-2.195 | 0.657 | 0.934 | 0.354-2.465 | 0.890 |
| CD11c^-^CD123^+^ CD80^+^ | 1.001 | 0.936-1.070 | 0.983 | 0.981 | 0.919-1.047 | 0.566 |
| CD45^+^ Lin-CD14^-^ HLA-DR^lo-^ CD11b^+^ CD33^+^ | 7.903 | 1.08-57.474 | **0.041** | 7.778 | 0.947-63.859 | 0.056 |
| CD45^+^ CD14^+^ HLA-DR^low^CD11b^+^ CD15^+^ | 0.948 | 0.494-1.820 | 0.873 | 0.747 | 0.399-1.399 | 0.362 |
| CD45^+^ CD14^-^ CD15^+^ CD11b+ | 1.004 | 0.972-1.037 | 0798 | 1.012 | 0.977-1.048 | 0.505 |
| Arginase-1 activity | 1.105 | 0.880-1.386 | 0.391 | 0.989 | 0.796-1.229 | 0.920 |
| Kyn/tryp ratio | 1.005 | 1.000-1.010 | 0.075 | 1.003 | 0.998-1.008 | 0.242 |
| sPD-1 | 0.995 | 0.977-1.012 | 0.549 | 0.989 | 0.970-1.007 | 0.232 |
| sPD-L1 | 1.019 | 0.999-1.040 | 0.065 | 0.998 | 0.979-1.018 | 0.863 |
| sPD-L2 | 1.000 | 1.000-1.000 | **0.036** | 1.000 | 1.000-1.000 | 0.149 |

CD, cluster of differentiation; HLA-DR, human leukocyte antigen –DR isotype; CTLA4, Cytotoxic T-Lymphocyte Associated Protein 4; ICOS, Inducible T Cell Co-stimulator; PD-1, Programmed Death Receptor 1; PD-L1, programmed death ligand-1; Tim3, T cell immunoglobulin and mucin domain-containing protein 3, OS; overall survival; PFS, progression free survival; CI, confidence interval; HR, hazard ratio

**Supplementary Table 8: Univariate Cox Regression results for OS and PFS for clinical variables**

| **Clinical variable** | **OS** | | | **PFS** | | |
| --- | --- | --- | --- | --- | --- | --- |
|  | **HR** | **CI (95%)** | **p-value** | **HR** | **CI (95%)** | **p-value** |
| Tumor types | 1.124 | 0.546-2.314 | 0.751 | 1.020 | 0.525-1.981 | 0.954 |
| Tumor grade | 1.454 | 0.803-2.631 | 0.217 | 1.118 | 0.656-1.905 | 0.682 |

OS; overall survival; PFS, progression free survival; CI, confidence interval; HR, hazard ratio

**Supplementary Table 9: Multivariate Cox Regression results for OS and PFS**

| **Endpoint** | **Marker/variable** | **Cell type** | **HR** | **95% CI** | **P-value** |
| --- | --- | --- | --- | --- | --- |
| OS | Grade | clinical variable | 1.701 | 0.752-3.848 | 0.202 |
| OS | Tumor type | clinical variable | 1.451 | 0.469-4.483 | 0.518 |
| OS | CD4^+^CD69^+^CD137^+^ | T helper cells | 1.053 | 0.514-2.156 | 0.888 |
| OS | CD4^+^CTLA4^+^PD-1^+^ | T helper cells | 2.036 | 1.191-3.481 | 0.009 |
| OS | CD56^+^CD16^+^CD69^+^ | CD56^+^CD16^+^CD69^+^ NK cells | 1.048 | 0.756-1.452 | 0.780 |
| OS | CD56^+^CD16^+^CD161^+^ | CD56^+^CD16^+^CD161^+^ NK cells | 1.092 | 0.968-1.231 | 0.152 |
| OS | CD56^-^CD16^+^ CD69^+^ | CD56-CD16+ CD69+ NK cells | 1.335 | 0.900-1.980 | 0.151 |
| OS | CD56^brigh^t^+^CD69^+^ | CD56^bright+^CD69^+^ NK cells | 0.932 | 0.8501.023 | 0.138 |
| OS | CD45^+^ Lin-CD14^-^ HLA-DR^lo-^ CD11b^+^ CD33^+^ | Early-stage MDSC | 4.898 | 0.370-64.767 | 0.228 |
| OS | sPD-L2 | Soluble PD-L2 | 1.000 | 1.000-1.000 | 0.089 |
| PFS | Grade | clinical variable | 0.994 | 0.545-1.812 | 0.985 |
| PFS | Tumor type | clinical variable | 1.068 | 0.495-2.305 | 0.866 |
| PFS | CD4^+^CD69^+^ | T helper cells | 1.174 | 0.897-1.536 | 0.242 |
| PFS | CD4^+^CD69^+^CD137^+^ | T helper cells | 1.437 | 0.604-3.421 | 0.412 |
| PFS | CD4^+^Tim3^+^ICOS^+^ | T helper cells | 1.164 | 0.912-1.486 | 0.233 |
| PFS | CD4^+^CTLA4^+^PD-1^+^ | T helper cells | 0.988 | 0.646-1.511 | 0.956 |
| PFS | CD56^+^CD16^+^CD69^+^ | CD56+CD16+CD69+ NK cells | 0.881 | 0.612-1.268 | 0.495 |
| PFS | CD56^+^CD16^+^CD161^+^ | CD56^+^CD16^+^CD161^+^ NK cells | 1.105 | 1.004-1.217 | 0.041 |
| PFS | CD56^-^CD16^+^ CD69^+^ | CD56^-^CD16^+^ CD69^+^ NK cells | 1.19 | 0.919-1.545 | 0.187 |
| PFS | CD56^bright+^CD69^+^ | CD56^bright+^CD69^+^ NK cells | 1.019 | 0.930-1.116 | 0.692 |

CD, cluster of differentiation; HLA-DR, human leukocyte antigen –DR isotype; CTLA4, Cytotoxic T-Lymphocyte Associated Protein 4; ICOS, Inducible T Cell Co-stimulator; PD-1, Programmed Death Receptor 1; PD-L1, programmed death ligand-1; Tim3, T cell immunoglobulin and mucin domain-containing protein 3, OS; overall survival; PFS, progression free survival; NK natural killer; MDSC, myeloid-derived suppressor cells; CI, confidence interval; HR, hazard ratio; OS, overall survival; PFS, progression free survival

**Supplementary Table 10: Multivariate Cox Regression analysis of prognostic value of combined score adjusted for clinical variables**

| **Variable** | **OS** | | | **PFS** | | |
| --- | --- | --- | --- | --- | --- | --- |
|  | **HR** | **CI (95%)** | **p-value** | **HR** | **CI (95%)** | **p-value** |
| Combined score | 19.040 | 2.572-140-957 | 0.004 | 7.079 | 2.613-19.176 | **<0.000** |
| Tumor grade | 1.176 | 0.678-2.038 | 0.564 | 0.893 | 0.536-1.486 | 0.662 |
| Tumor type | 0.998 | 0.478-2.085 | 0.995 | 0.859 | 0.438-1.686 | 0.659 |

CI, confidence interval; HR, hazard ratio; OS, overall survival; PFS, progression free survival

**Supplementary Table 11: Significant changes in immune cells and soluble**

**mediators in screening vs. Week 7 blood samples in non-responders**

| **Cell marker** | **Change in non-responders** | **P-value** |
| --- | --- | --- |
| Kyn/tryp | Increased | 0.0054 |
| sPD-1 | Increased | 0.0005 |
| Myeloid DCs | Increased | 0.0479 |
| CD80 in Plasmacytoid DCs | Decreased | 0.0345 |
| Mononuclear MDSCs | Increased | 0.0315 |
| CD56^+^CD16^+^CD161^+^ NK cells | Increased | 0.0101 |
| CD56^+^CD16^+^HLA-DR NK cells | Increased | 0.0146 |
| CD56^-^CD16^+^CD69^+^ NK cells | Increased | 0.0351 |
| CD56-CD16+HLA-DR NK  cells | Increased | 0.0008 |
| CD56^bright+^HLA-DR NK cells | Increased | 0.0335 |
| CD69 in helper T cells | Increased | 0.0026 |
| CD137 in helper T cells | Increased | 0.0052 |
| HLA-DR in helper T cells | Increased | 0.0007 |
| CD69^+^HLA-DR in helper T cells | Increased | 0.0013 |
| CD137^+^CD69 in helper T cells | Increased | 0.0004 |
| CD137^+^HLA-DR in helper T cells | Increased | 0.0010 |
| CTLA4 in helper T cells | Increased | 0.0010 |
| ICOS in helper T cells | Increased | 0.0026 |
| ICOS^+^CTLA4 in helper T cells | Increased | 0.0013 |
| Tim3^+^CTLA4 in helper T cells | Increased | 0.0027 |
| Tim3^+^ICOS in helper T cells | Increased | 0.0008 |
| CD69 in cytotoxic T cells | Increased | 0.0004 |
| CD137 in cytotoxic T cells | Increased | 0.0120 |
| HLA-DR in cytotoxic T cells | Increased | 0.0017 |
| CD69+HLA-DR in cytotoxic T cells | Increased | 0.0007 |
| CD137^+^CD69 in cytotoxic T cells | Increased | 0.0089 |
| CD137^+^HLA-DR in cytotoxic T cells | Increased | 0.0006 |
| ICOS in cytotoxic T cells | Increased | 0.0024 |
| Tim3 in cytotoxic T cells | Increased | 0.0126 |
| Tim3^+^ICOS in cytotoxic T cells | Increased | 0.0005 |
| Regulatory T cells | Increased | 0.0094 |
| Cytotoxic T cells | Decreased | 0.0161 |
| CD56^+^CD16^+^ NK cells | Decreased | 0.0039 |

CD. cluster of differentiation; HLA-DR. human leukocyte antigen –DR isotype; MDSC. myeloid-derived suppressor cell; DC. dendritic cell; NK. natural killer cell; CTLA4. Cytotoxic T-Lymphocyte Associated Protein 4; ICOS. Inducible T Cell Co-stimulator; PD-1. Programmed Death Receptor 1; PD-L1. programmed death ligand-1; Tim3. T cell immunoglobulin and mucin domain-containing protein 3

**Supplementary Table 12: Significant changes in immune cells and soluble**

**mediators in screening vs. EOT blood samples in non-responders**

| **Cell marker** | **Change in non-responders** | **P-value** |
| --- | --- | --- |
| sPD-1 | Increased | 0.0026 |
|  |  |  |
| Early stage MDSCs | Increased | 0.0303 |
|  |  |  |
| CD56^+^CD16^+^CD69 NK cells | Increased | 0.0052 |
| CD56^+^CD16^+^CD161 NK cells | Increased | 0.0052 |
|  |  |  |
|  |  |  |
| CD56^bright+^CD69 NK cells | Increased | 0.0180 |
|  |  |  |
| CD56^bright+^CD161 NK cells | Increased | 0.0052 |
| CD69 in helper T cells | Increased | 0.0041 |
| CD137 in helper T cells | Increased | 0.0172 |
| HLA-DR in helper T cells | Increased | 0.0175 |
| CD69^+^HLA-DR in helper T cells | Increased | 0.0135 |
|  |  |  |
| CD137^+^HLA-DR in helper T cells | Increased | 0.0034 |
| CTLA4 in helper T cells | Increased | 0.0044 |
| ICOS in helper T cells | Increased | 0.0342 |
| ICOS^+^CTLA4 in helper T cells | Increased | 0.0369 |
| Tim3^+^CTLA4 in helper T cells | Increased | 0.0189 |
| CD69 in cytotoxic T cells | Increased | 0.0069 |
| HLA-DR in cytotoxic T cells | Increased | 0.0315 |
| CD69^+^HLA-DR in cytotoxic T cells | Increased | 0.0373 |
| CD137^+^CD69 in cytotoxic T cells | Increased | 0.0356 |
| CD137^+^HLA-DR in cytotoxic T cells | Increased | 0.0147 |
| ICOS in cytotoxic T cells | Increased | 0.0160 |
|  |  |  |
| Tim3^+^ICOS in cytotoxic T cells | Increased | 0.0039 |
| Plasmacytoid DCs | Decreased | 0.0465 |

CD. cluster of differentiation; HLA-DR. human leukocyte antigen –DR isotype; MDSC.

myeloid-derived suppressor cell; DC. dendritic cell; NK. natural killer cell; CTLA4. Cytotoxic T-Lymphocyte Associated Protein 4; ICOS. Inducible T Cell Co-stimulator; PD-1. Programmed Death Receptor 1; PD-L1. programmed death ligand-1; Tim3. T cell immunoglobulin and mucin domain-containing protein 3

**Supplementary Table 13: Changes in immune cells and soluble mediators in cervical and endometrial cancer at screening vs. Week 7**

| Cell/Marker | Cervical Cancer | | | | | Endometrial Cancer | | | | |
| --- | --- | --- | --- | --- | --- | --- | --- | --- | --- | --- |
|  | Screening | | Week 7 | | P-value | Screening | | Week 7 | | P-value |
|  | Mean | Median | Mean | Median |  | Mean | Median | Mean | Median |  |
| CD3^+^ | 29.87 | 27.00 | 31.60 | 31.70 | 0.6791 | 31.12 | 26.35 | 28.14 | 27.05 | 0.6886 |
| CD4^+^ | 16.08 | 15.60 | 18.97 | 19.10 | 0.4761 | 17.30 | 18.50 | 15.12 | 15.80 | 0.4330 |
| CD4^+^CD69^+^ | 3.029 | 2.470 | 5.004 | 4.720 | 0.0552 | 3.233 | 2.620 | 6.592 | 6.105 | **0.0122** |
| CD4^+^CD137^+^ | 2.436 | 2.230 | 4.128 | 4.200 | **0.0078** | 3.149 | 2.955 | 5.076 | 4.075 | **0.0397** |
| CD4^+^HLA-DR^+^ | 5.512 | 4.630 | 7.983 | 9.170 | 0.0527 | 5.038 | 4.490 | 8.281 | 7.710 | **0.0256** |
| CD4^+^CD69^+^HLA-DR^+^ | 2.555 | 2.130 | 5.949 | 5.970 | **0.0012** | 3.189 | 2.750 | 6.088 | 5.320 | **0.0095** |
| CD4^+^CD69^+^CD137^+^ | 1.015 | 0.8200 | 2.035 | 0.9700 | 0.2480 | 0.8244 | 0.8000 | 1.687 | 1.290 | **0.0050** |
| CD4^+^CD137^+^HLA-DR^+^ | 2.623 | 2.380 | 5.641 | 5.130 | **0.0050** | 3.027 | 2.650 | 6.067 | 5.405 | **0.0050** |
| CD4^+^CTLA4^+^ | 2.302 | 2.050 | 3.408 | 2.720 | **0.0174** | 2.352 | 1.810 | 3.878 | 4.100 | **0.0180** |
| CD4^+^ICOS^+^ | 8.022 | 8.050 | 11.10 | 10.80 | **0.0457** | 8.523 | 7.925 | 11.50 | 10.50 | **0.0066** |
| CD4^+^Tim3+ | 3.208 | 2.500 | 4.604 | 3.500 | 0.0505 | 4.620 | 2.630 | 3.989 | 3.405 | 0.9010 |
| CD4^+^Tim3^+^ICOS^+^ | 5.165 | 4.920 | 7.411 | 7.160 | **0.0200** | 4.648 | 4.630 | 7.374 | 6.680 | **0.0066** |
| CD4^+^CTLA4^+^ICOS^+^ | 1.080 | 1.000 | 1.744 | 1.570 | **0.0095** | 1.242 | 0.9950 | 2.078 | 1.735 | **0.0408** |
| CD8^+^ | 11.18 | 10.30 | 9.938 | 9.260 | 0.2955 | 12.53 | 11.70 | 10.13 | 7.900 | 0.4100 |
| CD8^+^CD69^+^ | 6.245 | 7.020 | 11.50 | 9.340 | **0.0041** | 9.873 | 6.690 | 15.55 | 11.85 | **0.0050** |
| CD8^+^CD137^+^ | 3.734 | 3.330 | 5.536 | 3.890 | 0.0822 | 4.821 | 4.015 | 5.795 | 4.640 | 0.2601 |
| CD8^+^HLA-DR^+^ | 6.388 | 5.150 | 11.08 | 9.780 | **0.0223** | 5.998 | 5.565 | 12.17 | 10.90 | **0.0025** |
| CD8^+^CD69^+^CD137^+^ | 4.995 | 4.590 | 5.649 | 5.530 | 0.4643 | 6.794 | 3.175 | 10.20 | 5.770 | **0.0016** |
| CD8^+^CD137^+^HLA-DR^+^ | 3.779 | 3.010 | 7.782 | 6.410 | **0.0068** | 4.140 | 3.440 | 8.522 | 7.585 | **0.0020** |
| CD8^+^Tim3^+^ | 2.702 | 1.840 | 5.161 | 4.470 | **0.0176** | 3.234 | 1.790 | 4.623 | 4.110 | 0.1565 |
| CD8^+^CTLA4^+^ | 3.288 | 2.510 | 4.108 | 3.150 | 0.3656 | 5.511 | 2.190 | 4.899 | 4.435 | 0.8847 |
| CD8^+^ICOS^+^ | 5.826 | 4.150 | 9.020 | 8.820 | **0.0133** | 6.557 | 6.305 | 8.717 | 8.910 | **0.0478** |
| CD8^+^Tim3^+^CTLA4^+^ | 0.8965 | 0.6700 | 1.554 | 1.190 | 0.0938 | 1.901 | 0.6600 | 1.489 | 1.520 | 0.7615 |
| CD8^+^Tim3^+^ICOS^+^ | 2.062 | 1.810 | 4.515 | 3.720 | **0.0025** | 1.895 | 1.785 | 3.620 | 3.215 | **0.0016** |
| CD8^+^CTLA4^+^ICOS^+^ | 1.298 | 1.120 | 1.995 | 1.510 | 0.0780 | 2.452 | 1.345 | 2.224 | 1.970 | 0.9661 |
| CD4^+^CD25^+^FOXP3^+^ | 0.5129 | 0.4000 | 1.017 | 0.9100 | **0.0209** | 0.6600 | 0.5550 | 0.9539 | 0.9550 | **0.0418** |
| CD56^+^CD16^+^ | 7.945 | 6.480 | 6.929 | 5.050 | 0.2490 | 8.070 | 7.790 | 5.696 | 4.580 | **0.0133** |
| CD56^+^CD16^+^CD69^+^ | 1.249 | 0.9900 | 2.165 | 1.570 | 0.1739 | 2.295 | 1.380 | 2.251 | 1.685 | 0.3777 |
| CD56^+^CD16^+^CD161^+^ | 2.432 | 1.750 | 4.193 | 4.580 | **0.0106** | 3.422 | 1.590 | 4.555 | 3.155 | 0.0822 |
| CD56^+^CD16^+^HLA-DR^+^ | 12.48 | 12.90 | 21.99 | 19.30 | 0.0790 | 13.39 | 12.90 | 21.23 | 15.20 | 0.06575 |
| CD56^-^CD16^+^ | 3.489 | 3.450 | 4.093 | 4.150 | 0.5943 | 4.152 | 3.390 | 3.727 | 3.765 | 0.8522 |
| CD56^-^CD16^+^ CD69+ | 1.654 | 1.140 | 2.574 | 2.520 | **0.0305** | 2.155 | 1.625 | 2.514 | 2.205 | 0.2111 |
| CD56^-^CD16^+^ CD161^+^ | 5.034 | 4.570 | 5.447 | 4.280 | 0.6643 | 7.336 | 3.915 | 4.726 | 4.500 | 0.8176 |
| CD56^-^CD16^+^ HLA-DR^+^ | 39.31 | 44.80 | 63.71 | 65.10 | **0.0040** | 46.16 | 47.85 | 57.05 | 63.15 | 0.0780 |
| CD56^bright^ | 1.379 | 0.9900 | 1.392 | 1.200 | 0.9359 | 1.086 | 1.005 | 1.516 | 1.305 | 0.3319 |
| CD56^bright+^CD69^+^ | 3.572 | 2.630 | 5.905 | 3.960 | 0.1423 | 6.543 | 4.450 | 4.337 | 4.375 | 0.4021 |
| CD56^bright^+CD161+ | 9.475 | 9.110 | 9.082 | 8.640 | 0.3840 | 10.61 | 10.40 | 9.400 | 9.140 | 0.4778 |
| CD56^bright+^HLA-DR^+^ | 51.23 | 55.85 | 53.34 | 49.80 | 0.6316 | 52.10 | 50.95 | 62.93 | 67.65 | 0.1028 |
| CD11c^+^CD123^-^ | 3.340 | 2.490 | 4.181 | 3.190 | 0.0971 | 3.956 | 4.100 | 4.929 | 4.430 | 0.2369 |
| CD11c^+^CD123^-^ CD80^+^ | 4.063 | 4.050 | 3.819 | 3.860 | 0.6155 | 3.789 | 3.350 | 3.537 | 3.230 | 0.9166 |
| CD11c^-^CD123^+^ | 0.4488 | 0.3100 | 0.3382 | 0.3200 | 0.2511 | 0.4364 | 0.3450 | 0.4281 | 0.4350 | 0.6732 |
| CD11c^-^CD123^+^ CD80^+^ | 7.761 | 6.800 | 7.465 | 6.020 | 0.6580 | 8.856 | 6.480 | 6.162 | 5.290 | 0.1239 |
| CD45^+^ Lin-CD14^-^ HLA-DR^lo-^ CD11b^+^ CD33^+^ | 0.1939 | 0.1845 | 0.2874 | 0.1875 | 0.5678 | 0.2636 | 0.2024 | 0.2893 | 0.2918 | 0.4857 |
| CD45^+^ CD14^+^ HLA-DR^low^CD11b^+^ CD15^+^ | 0.9779 | 0.7820 | 1.157 | 1.060 | 0.6794 | 1.049 | 0.9080 | 1.262 | 1.355 | 0.3273 |
| CD45^+^ CD14^-^ CD15^+^ CD11b^+^ | 65.70 | 67.20 | 66.74 | 71.60 | 0. 6297 | 68.71 | 71.80 | 70.51 | 71.70 | 0.7062 |
| Arginase-1 activity | 3.012 | 2.490 | 2.688 | 0.9999 | >0.9999 | 2.551 | 2.180 | 2.618 | 2.273 | 0.7661 |
| Kyn/tryp ratio | 67.33 | 56.88 | 145.7 | 130.1 | 0.0094 | 88.74 | 73.93 | 124.1 | 94.60 | 0.3846 |
| sPD-1 | 8.686 | 5.285 | 461.4 | 296.9 | **0.0016** | 17.42 | 5.875 | 541.9 | 416.9 | **0.0012** |
| sPD-L1 | 19.53 | 10.04 | 25.21 | 13.88 | 0.2640 | 22.33 | 14.58 | 29.85 | 16.42 | 0.1376 |
| sPD-L2 | 8441 | 7889 | 8831 | 8470 | 0.2579 | 8736 | 8054 | 8419 | 7703 | 0.7531 |

CD, cluster of differentiation; HLA-DR, human leukocyte antigen –DR isotype; CTLA4, Cytotoxic T-Lymphocyte Associated Protein 4; ICOS, Inducible T Cell Co-stimulator; PD-1, Programmed Death Receptor 1; PD-L1, programmed death ligand-1; Tim3, T cell immunoglobulin and mucin domain-containing protein 3.

**Supplementary Table 14: Changes in immune cells and soluble mediators in cervical and endometrial cancer at screening vs. EOT**

| Cell/Marker | Cervical Cancer | | | | | Endometrial Cancer | | | | |
| --- | --- | --- | --- | --- | --- | --- | --- | --- | --- | --- |
|  | Screening | | EOT | | P-value | Screening | | EOT | | P-value |
|  | Mean | Median | Mean | Median |  | mean | median | mean | median |  |
| CD3^+^ | 33.30 | 28.50 | 34.94 | 32.40 | 0.7780 | 28.83 | 24.80 | 25.53 | 29.70 | 0.3320 |
| CD4^+^ | 16.66 | 16.50 | 18.17 | 16.70 | 0.4328 | 15.90 | 14.90 | 15.16 | 12.80 | 0.8175 |
| CD4^+^CD69^+^ | 3.096 | 2.470 | 4.401 | 3.690 | 0.1302 | 2.604 | 2.050 | 6.815 | 6.460 | **0.0250** |
| CD4^+^CD137^+^ | 2.323 | 2.230 | 4.680 | 4.200 | **0.0333** | 3.248 | 3.000 | 4.886 | 4.400 | 0.2780 |
| CD4^+^HLA-DR^+^ | 5.985 | 4.670 | 6.514 | 5.140 | 0.4805 | 5.644 | 6.100 | 7.096 | 7.030 | 0.7020 |
| CD4^+^CD69^+^HLA-DR^+^ | 2.178 | 2.120 | 4.586 | 3.510 | **0.0433** | 3.707 | 3.430 | 8.715 | 5.390 | 0.2106 |
| CD4^+^CD69^+^CD137^+^ | 0.9633 | 0.8100 | 1.444 | 1.010 | 0.4682 | 0.9027 | 0.8500 | 2.846 | 1.830 | 0.0930 |
| CD4^+^CD137^+^HLA-DR^+^ | 2.401 | 2.260 | 4.605 | 3.110 | 0.0845 | 3.478 | 3.750 | 8.856 | 5.820 | 0.0544 |
| CD4^+^CTLA4^+^ | 2.359 | 1.820 | 3.885 | 3.000 | 0.1278 | 2.368 | 1.820 | 3.374 | 2.720 | 0.2075 |
| CD4^+^ICOS^+^ | 7.239 | 6.880 | 11.46 | 10.80 | 0.1220 | 9.412 | 9.970 | 9.882 | 9.190 | 0.7971 |
| CD4^+^Tim3^+^ | 2.660 | 2.280 | 4.026 | 3.150 | 0.3122 | 5.482 | 2.650 | 4.773 | 4.350 | 0.8841 |
| CD4^+^Tim3^+^ICOS^+^ | 4.852 | 4.920 | 6.393 | 5.050 | 0.1954 | 5.442 | 5.560 | 6.220 | 6.100 | 0.6075 |
| CD4^+^CTLA4^+^ICOS^+^ | 0.9573 | 0.8200 | 1.513 | 1.360 | **0.0250** | 1.247 | 1.305 | 1.658 | 1.380 | 0.6212 |
| CD8^+^ | 13.53 | 13.50 | 14.20 | 12.50 | 0.8841 | 10.14 | 5.910 | 8.524 | 6.720 | 0.4638 |
| CD8^+^CD69^+^ | 5.778 | 7.020 | 9.807 | 9.020 | 0.0853 | 11.65 | 7.040 | 19.11 | 17.20 | **0.0350** |
| CD8^+^CD137^+^ | 3.852 | 3.750 | 5.003 | 4.660 | 0.3431 | 5.410 | 4.130 | 5.904 | 6.590 | 0.5890 |
| CD8^+^HLA-DR^+^ | 6.683 | 5.150 | 12.78 | 7.420 | 0.1167 | 6.308 | 6.370 | 10.74 | 12.30 | 0.1500 |
| CD8^+^CD69^+^CD137^+^ | 4.458 | 4.090 | 5.435 | 4.600 | 0.2633 | 9.268 | 4.270 | 15.95 | 10.00 | **0.0166** |
| CD8^+^CD137^+^HLA-DR^+^ | 3.293 | 2.600 | 5.051 | 3.610 | 0.2277 | 4.611 | 4.970 | 10.12 | 9.060 | **0.0425** |
| CD8^+^Tim3^+^ | 2.285 | 1.640 | 4.003 | 3.660 | 0.1953 | 3.772 | 2.570 | 5.652 | 5.000 | 0.1976 |
| CD8^+^CTLA4^+^ | 2.866 | 1.790 | 3.811 | 2.830 | 0.2819 | 7.110 | 7.440 | 5.508 | 5.350 | 0.5706 |
| CD8^+^ICOS^+^ | 5.241 | 3.980 | 9.348 | 5.360 | 0.0938 | 7.413 | 7.330 | 9.680 | 9.160 | 0.0845 |
| CD8^+^Tim3^+^CTLA4^+^ | 0.6127 | 0.3500 | 0.9373 | 0.7800 | 0.1336 | 2.400 | 2.060 | 2.550 | 1.310 | 0.8657 |
| CD8^+^Tim3^+^ICOS^+^ | 1.875 | 1.340 | 2.756 | 2.710 | 0.0775 | 2.020 | 1.920 | 5.177 | 4.320 | **0.0166** |
| CD8^+^CTLA4^+^ICOS^+^ | 0.9418 | 1.060 | 1.657 | 1.150 | 0.1729 | 3.233 | 2.140 | 2.866 | 1.800 | 0.7956 |
| CD4^+^CD25^+^FOXP3^+^ | 0.5218 | 0.3800 | 0.7755 | 0.7400 | 0.1150 | 0.5836 | 0.4500 | 1.038 | 0.9500 | 0.2081 |
| CD56^+^CD16^+^ | 9.027 | 8.350 | 6.191 | 5.420 | 0.1305 | 7.047 | 6.790 | 3.934 | 2.840 | 0.1982 |
| CD56^+^CD16^+^CD69^+^ | 1.396 | 1.430 | 3.579 | 3.625 | **0.0340** | 2.752 | 1.550 | 11.12 | 10.60 | **0.0125** |
| CD56^+^CD16^+^CD161^+^ | 2.153 | 1.675 | 7.800 | 6.530 | **0.0500** | 4.370 | 1.960 | 14.50 | 14.60 | **0.01**  **00** |
| CD56^+^CD16^+^HLA-DR^+^ | 12.09 | 12.65 | 15.88 | 8.845 | 0.8597 | 17.01 | 16.10 | 20.43 | 11.50 | 0.7780 |
| CD56^-^CD16^+^ | 3.410 | 3.510 | 3.170 | 2.430 | 0.6173 | 5.228 | 4.390 | 9.685 | 6.870 | 0.7610 |
| CD56^-^CD16^+^ CD69^+^ | 1.846 | 1.470 | 3.934 | 3.780 | 0.0960 | 2.310 | 1.680 | 5.178 | 5.270 | 0.1400 |
| CD56^-^CD16^+^ CD161^+^ | 4.593 | 4.180 | 7.852 | 6.600 | **0.0425** | 4.846 | 3.600 | 10.33 | 10.10 | 0.1872 |
| CD56^-^CD16^+^ HLA-DR^+^ | 38.36 | 40.30 | 44.49 | 56.90 | 0.4626 | 51.56 | 54.60 | 57.68 | 67.00 | 0.5901 |
| CD56^bright^ | 1.613 | 1.105 | 1.903 | 1.545 | 0.5168 | 1.181 | 1.060 | 1.849 | 1.450 | 0.2729 |
| CD56^bright+^CD69^+^ | 2.718 | 2.480 | 6.693 | 5.910 | **0.0350** | 7.318 | 4.160 | 20.94 | 12.40 | 0.1238 |
| CD56^bright+^CD161^+^ | 8.872 | 8.950 | 18.31 | 13.15 | 0.0896 | 11.41 | 12.00 | 18.29 | 17.10 | 0.0775 |
| CD56^bright+^HLA-DR^+^ | 48.86 | 47.00 | 49.15 | 44.25 | 0.98949 | 56.23 | 62.10 | 53.47 | 53.40 | 0.7776 |
| CD11c^+^CD123^-^ | 3.377 | 2.430 | 3.169 | 2.210 | 0.9999 | 4.875 | 5.310 | 6.228 | 6.480 | 0.4141 |
| CD11c^+^CD123^-^ CD80^+^ | 4.010 | 4.145 | 3.723 | 3.560 | 0.3181 | 4.488 | 4.010 | 5.435 | 3.800 | 1.0203 |
| CD11c^-^CD123^+^ | 0.3967 | 0.2900 | 0.2793 | 0.2450 | 0.2523 | 0.5564 | 0.3400 | 0.3975 | 0.4300 | 0.4122 |
| CD11c^-^CD123^+^ CD80+ | 8.151 | 8.380 | 10.80 | 9.860 | 0.3432 | 6.766 | 5.480 | 9.833 | 5.470 | 0.7591 |
| CD45^+^ Lin-CD14^-^ HLA-DR^lo-^ CD11b^+^ CD33^+^ | 0.1612 | 0.1773 | 0.3937 | 0.1838 | 0.2543 | 0.2392 | 0.1771 | 0.4063 | 0.1955 | 0.3963 |
| CD45^+^ CD14^+^ HLA-DR^low^CD11b^+^ CD15^+^ | 0.8765 | 0.7960 | 1.177 | 1.110 | 0.3865 | 1.352 | 1.300 | 1.187 | 1.330 | 0.4116 |
| CD45^+^ CD14^-^ CD15^+^ CD11b^+^ | 66.05 | 71.00 | 71.63 | 77.50 | 0.4170 | 70.32 | 74.90 | 74.23 | 74.10 | 0.5681 |
| Arginase-1 activity | 2.942 | 2.717 | 3.622 | 2.793 | 0.9358 | 2.197 | 1.983 | 3.476 | 3.352 | 0.3672 |
| Kyn/tryp ratio | 69.67 | 56.88 | 115.7 | 128.2 | **0.0685** | 101.3 | 89.28 | 125.5 | 84.50 | 0.8185 |
| sPD-1 | 4.605 | 3.505 | 710.5 | 512.2 | **0.0250** | 22.17 | 5.875 | 630.1 | 555.6 | **0.0250** |
| sPD-L1 | 14.32 | 10.04 | 27.51 | 11.87 | 0.6040 | 19.55 | 14.58 | 22.53 | 21.34 | 0.7415 |
| sPD-L2 | 7789 | 7889 | 10054 | 9597 | **0.0325** | 9113 | 8054 | 7863 | 7562 | 0.5763 |

CD, cluster of differentiation; HLA-DR, human leukocyte antigen –DR isotype; CTLA4, Cytotoxic T-Lymphocyte Associated Protein 4; ICOS, Inducible T Cell Co-stimulator; PD-1, Programmed Death Receptor 1; PD-L1, programmed death ligand-1; Tim3, T cell immunoglobulin and mucin domain-containing protein 3; EOT, end of treatment

# ***Supplementary methods***

**Gating strategy and immune cell subpopulations**

We developed the manual gating strategy for immunophenotyping of immune cells as indicated in Suppl. Fig 1- 4. Firstly. we identified single cells (FSC-H/FSC-A) followed by gating of live cells. T cells were defined as CD45^+^CD3^+^. helper T cells (CD4^+^ T cells) as CD45^+^CD3^+^CD4^+^CD8^-^. cytotoxic T cells (CD8^+^ T cells) as CD45^+^CD3^+^CD4^-^CD8^+^ and Treg as CD45^+^CD3^+^CD4^+^CD8^-^CD25^high^CD127^low^ FoxP3^+^. NK subgroups were defined as follows: CD56^+^CD16^+^ NK cells (CD45^+^CD3^-^CD56^+^CD16^+^). CD56^bright^CD16^+/-^NK cells (CD45^+^CD3^-^CD56^+^CD16^-^) and CD56^-^CD16^+^ NK cells (CD45^+^CD3^-^CD56^-^ CD16^+^). DC subgroups were defined as follows: myeloid DC (CD45^+^CD14^-^HLA-DR^+^CD11c^+^CD123^-^) and plasmacytoid DC (CD45^+^CD14^-^HLA-DR^+^CD11c^-^CD123^+^). MDSCs were defined as follow: PMN-MDSC (CD45^+^CD14^-^CD15^+^CD11b^+^). M-MDSC (CD45^+^CD14^+^HLA-DR^low^CD11b^+^CD15^+^) and early stage (e-MDSC) (CD45^+^Lin-CD14^-^HLA-DR^lo^CD11b^+^CD33^+^).

# **
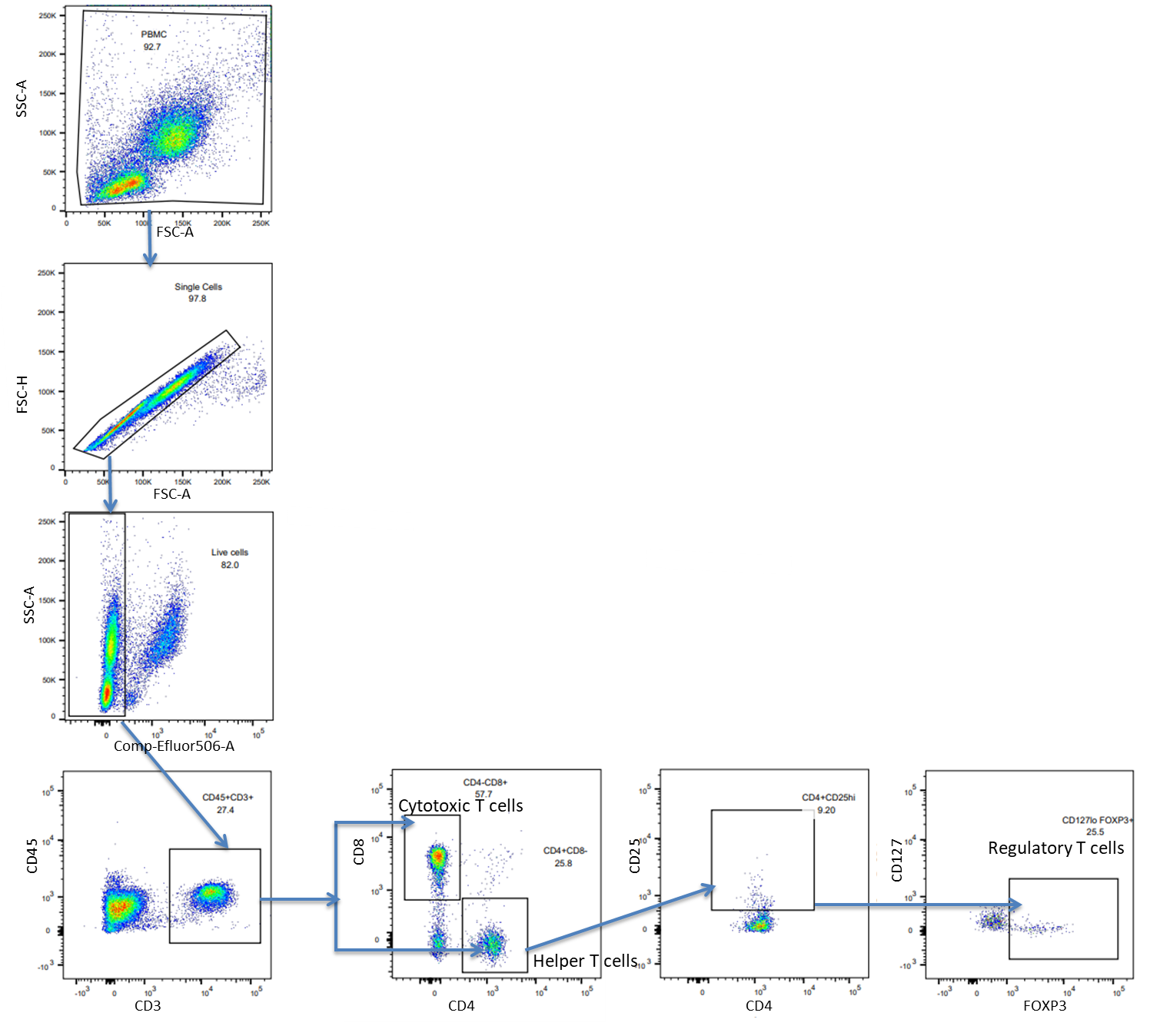
*Supplementary Figures***

**Suppl. Fig. 1. Representative manual gating strategy for phenotyping T cell subpopulations:** Cytotoxic T cells (CD45^+^CD3^+^CD4^-^CD8^+^). helper T cells (CD45^+^CD3^+^CD4^+^CD8^-^) and Regulatory T cells (CD45^+^CD3^+^CD4^+^CD8^-^CD25^high^CD127^low^FoxP3^+^)

**
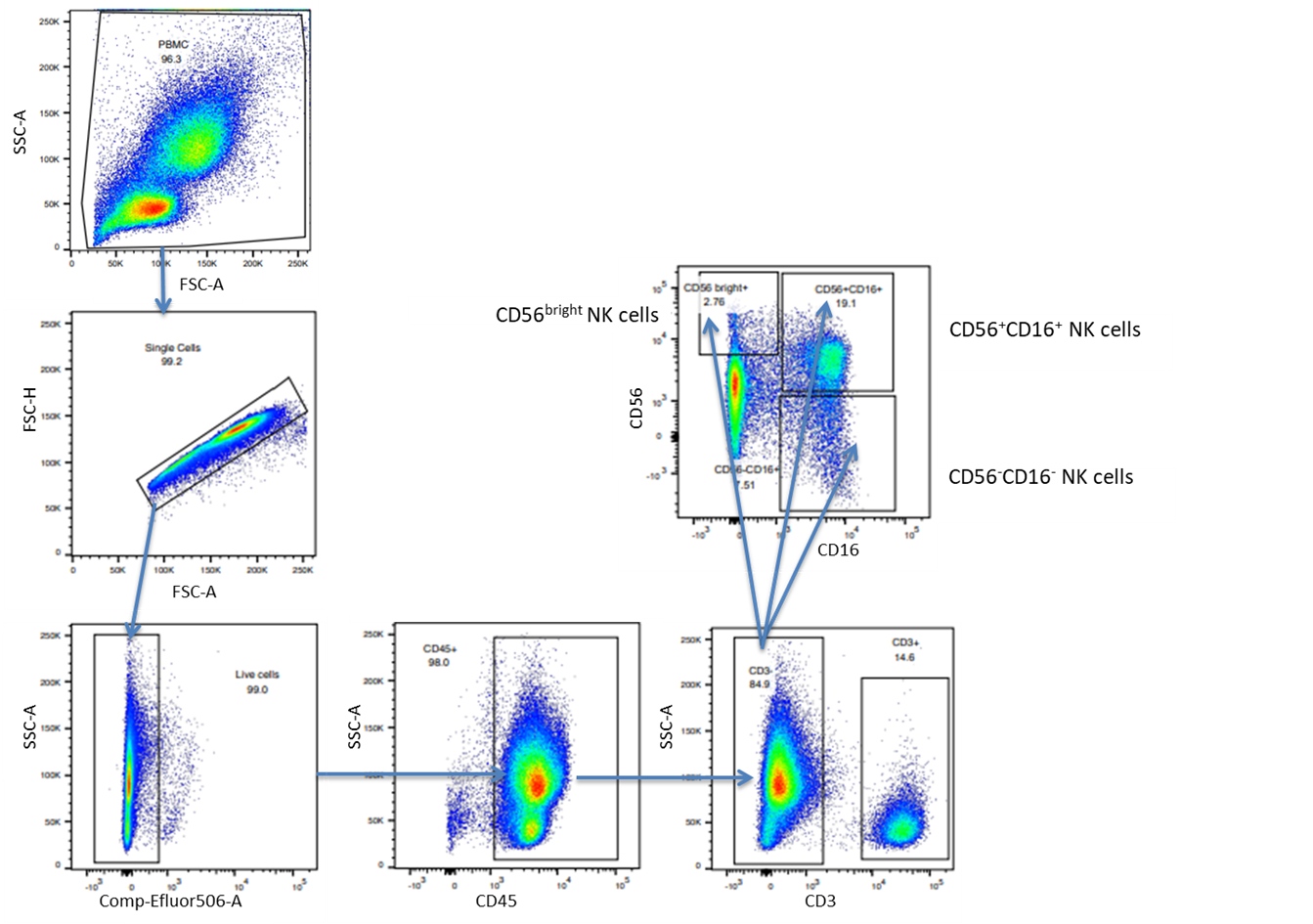
Suppl. Fig. 2. Representative manual gating strategy for phenotyping NK cell subpopulations:** CD56^+^CD16^+^ NK cells (CD45^+^ CD3^-^ CD56^+^ CD16^+^). CD56^bright^CD16^+/-^NK cells (CD45^+^ CD3^-^ CD56^+^ CD16^-^) and CD56^-^CD16^+^ NK cells (CD45^+^ CD3^-^ CD56^-^ CD16^+^).

**
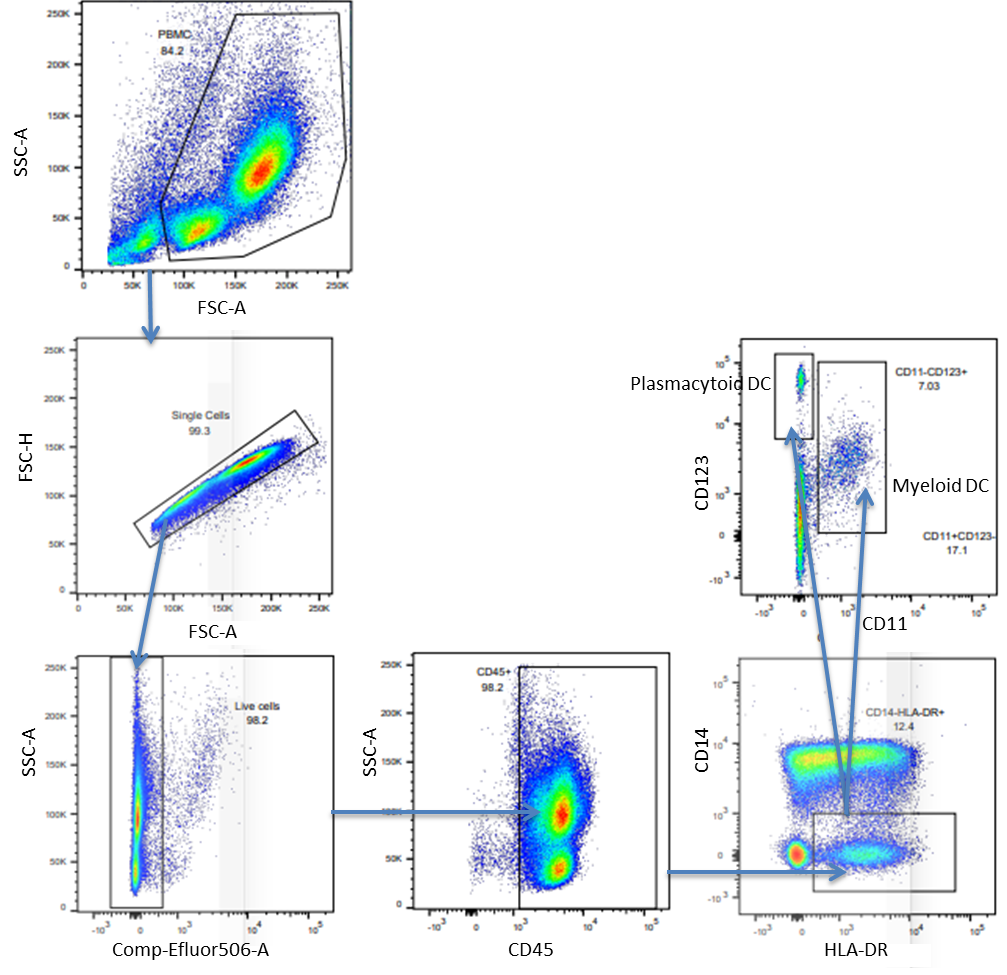
Suppl. Fig. 3. Representative manual gating strategy for phenotyping DC cell subpopulations:** Myeloid DC (CD45^+^ CD14^-^ HLA-DR^+^ CD11c^+^ CD123^-^) and plasmacytoid DC (CD45^+^ CD14^-^ HLA-DR^+^ CD11c^-^ CD123^+^).

**
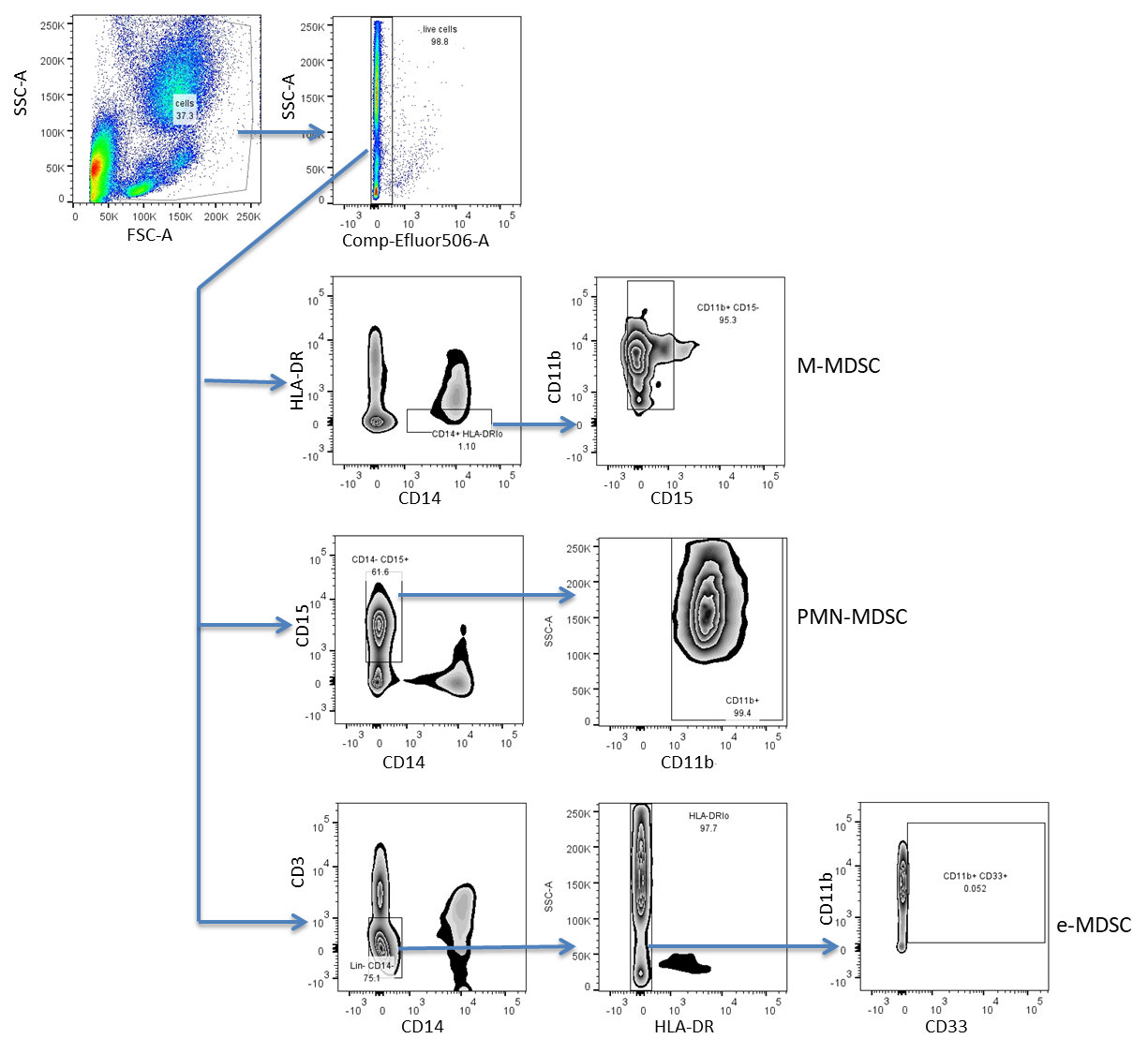
Suppl. Fig. 4. Representative manual gating strategy for phenotyping MDSC cell subpopulations:** PMN-MDSC (CD45^+^CD14^-^CD15^+^CD11b^+^). M-MDSC (CD45^+^CD14^+^HLA-DR^low^CD11b^+^CD15^+^) and early stage (e-MDSC) (CD45^+^Lin-CD14^-^HLA-DR^lo^CD11b^+^CD33^+^).

**
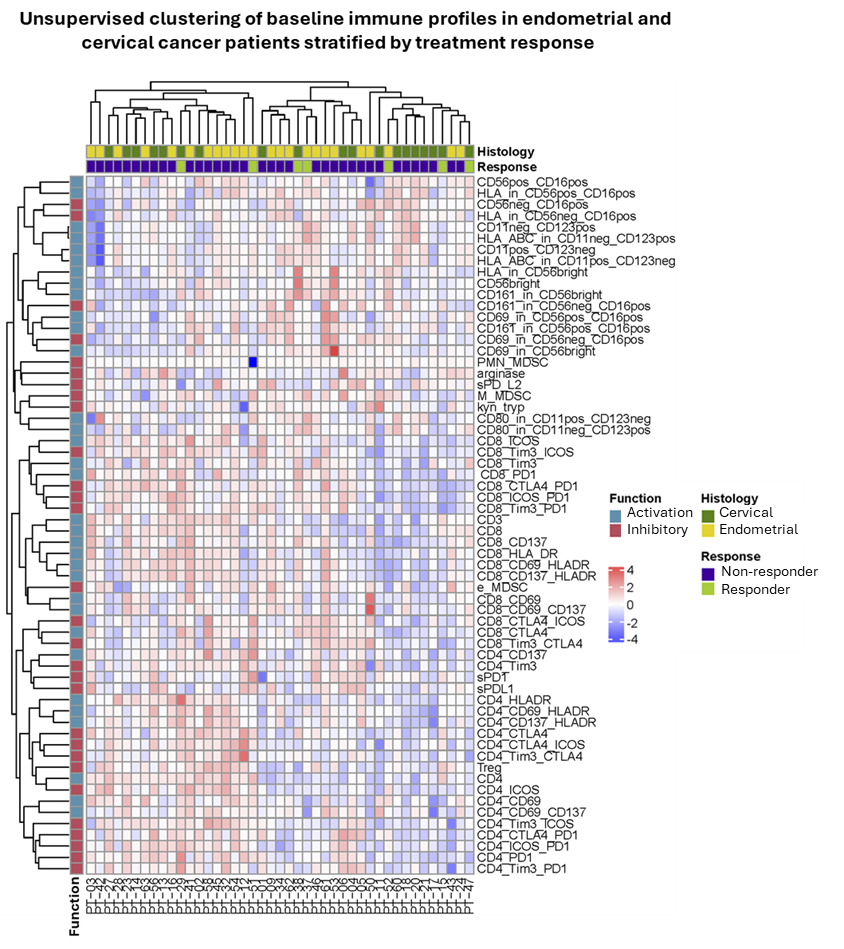
**


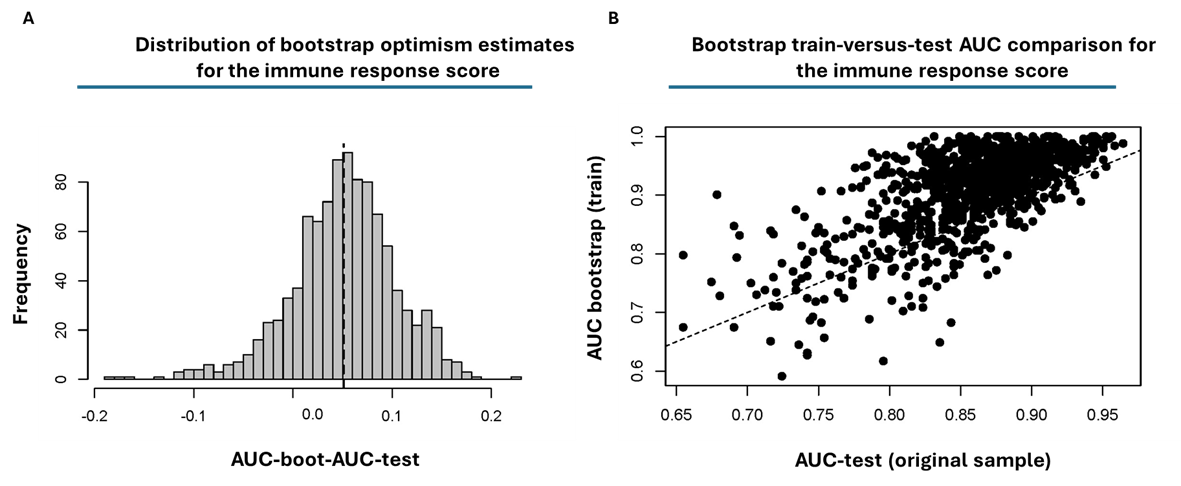
**Suppl. Fig. 5.** Rows correspond to individual immune cells and inflammatory mediators. while columns represent individual patient samples. Data was normalized across features. and unsupervised hierarchical clustering was applied to both rows and columns to reveal intrinsic patterns without prior class labeling. Colors indicate relative expression or abundance. with red representing higher levels and blue representing lower levels. **Suppl. Fig 6. Internal validation of the immune response score using bootstrap resampling:** **(A)** Histogram showing the distribution of optimism values obtained from bootstrap internal validation (1,000 resamples). The dashed line indicates mean optimism; positive values reflect overfitting and were used to adjust the model’s apparent AUC; **(B)** Scatter plot comparing AUCs from bootstrap samples (training) with those obtained by applying the same model-re-estimated cutoffs and score reconstruction-to the original cohort (test set). Each point represents a bootstrap iteration. The dashed diagonal indicates equal training and test performance (no optimism); points above it reflect overfitting, with vertical distance representing the magnitude of optimism.

**
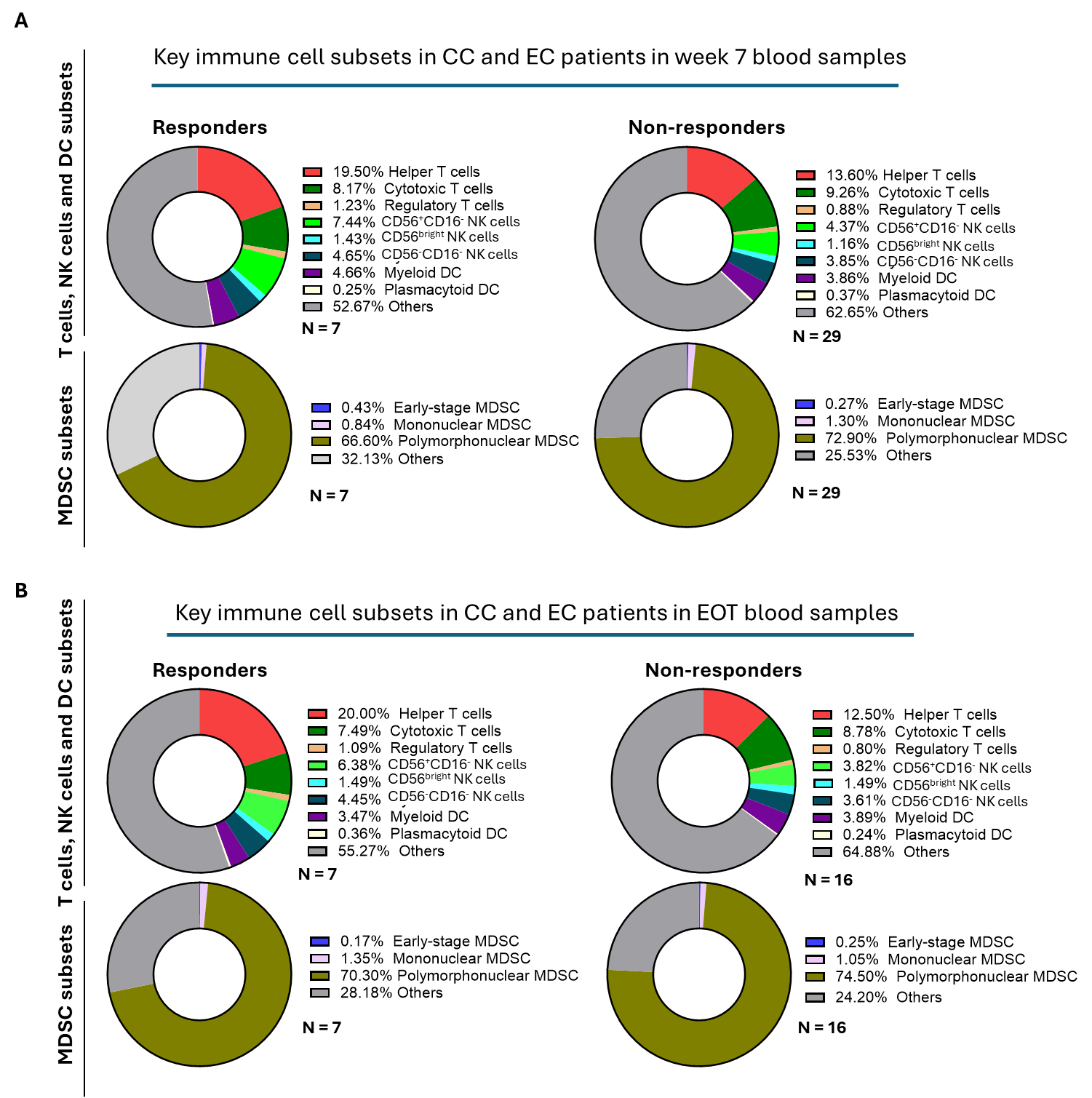
Suppl. Fig 7. Composition of key immune cell subsets in cervical and endometrial cancer patients during treatment:** **(A)** Pie charts showing percentage of T cell. NK cell and DC subsets in PBMCs and MDSC subsets in whole blood in responders and non-responders at week 7; **(B)** Pie charts showing percentage of T cell. NK cell and DC subsets in PBMCs and MDSC subsets in whole blood in responders and non-responders at EOT. Percentages represent median values of each subpopulation.

**
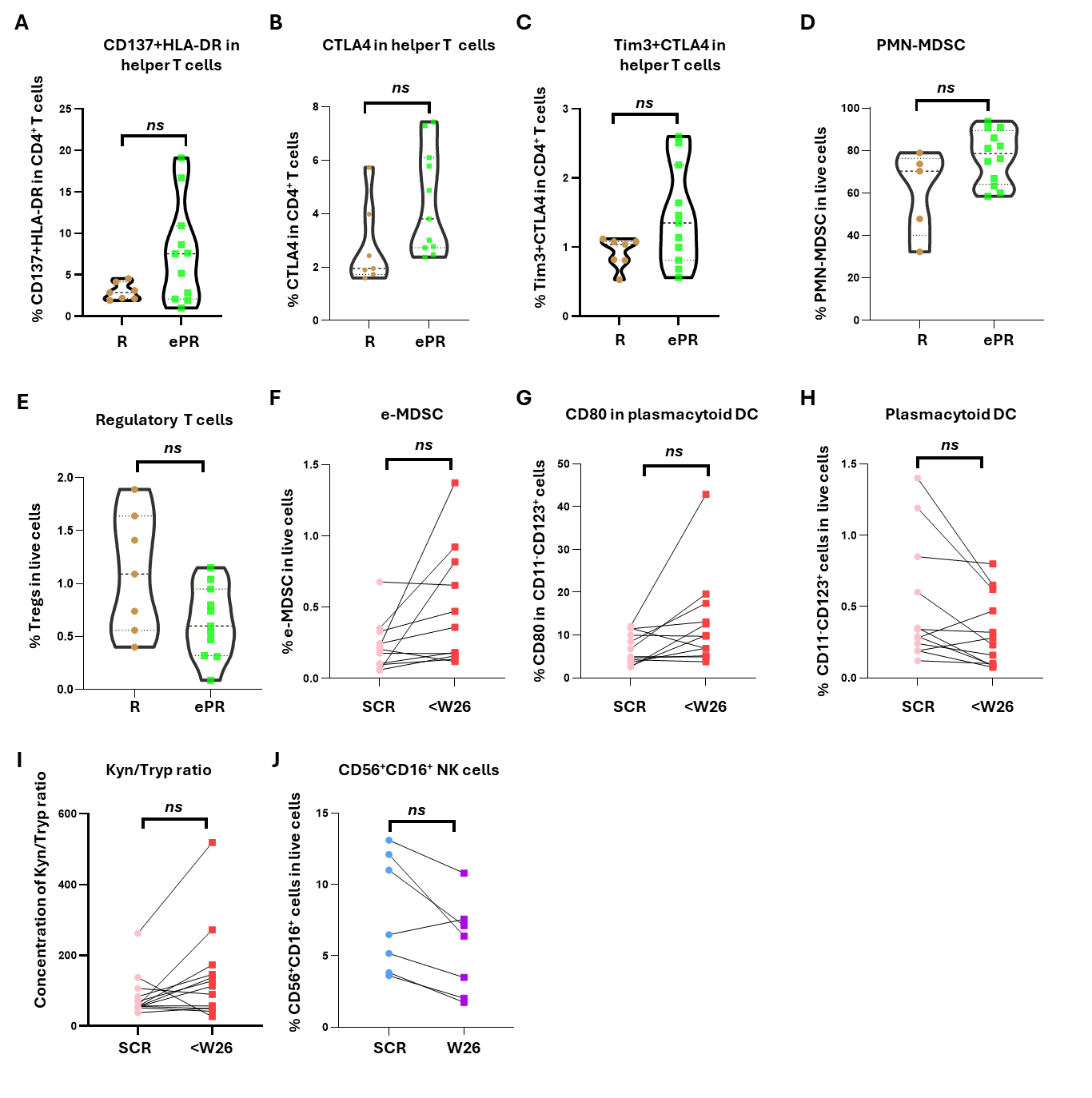
**

**Suppl. Fig 8. Immune profiling in patients with early disease progression and responders:** **(A-E)** Violin plots showing the percentage of **(A)** CD137+HLA-DR in helper T cells, **(B)** CTLA4 expression in helper T cells, **(C)** Tim3+CTLA4 in helper T cells, **(D)** PMN-MDSC and **(E)** regulatory T cells in responders (n = 7; brown) and early progressors (n = 13; green) at EOT (week 26 for responders and <week 26 for early progressors). Note: Panel **D** has 5 responders due to technical error. Significance difference was tested by Mann-Whitney, with multiple comparison using the Benjamini-Hochberg false discovery rate (FDR) procedure. **(F-I)** Line graphs showing the percentage of **(F)** e-MDSC, **(G)** CD80 expression in plasmacytoid DC, **(H)** plasmacytoid DC and **(I)** kyn/tryp ratio at screening (n = 13; pink) vs <Week 26 (n = 13; red). **(J)** Line graph showing the percentage of CD56^+^CD16^+^ NK cells in live cells at screening (n = 7; blue) vs Week 26 (n = 7; purple) in responders. Significance difference was tested by Wilcoxon- ranked test, with multiple comparison using the Benjamini-Hochberg false discovery rate (FDR) procedure. Significance difference was accepted at p ≤ 0.05. R: responders, ePR: early progressors, SCR: screening, W26: week 26.
